# The role of MiRNA-mediated tumor microenvironment in bone metastasis from a multi-omics perspective: cross-cancer mechanisms and clinical translation

**DOI:** 10.3389/fonc.2026.1775016

**Published:** 2026-02-26

**Authors:** Zhuo Gu, Shu-fen Zhu, Peng-fei Li

**Affiliations:** 1 Department of Orthopaedics, Inner Mongolia Autonomous Region People’s Hospital, Hohhot, Inner Mongolia, China; 2 Physical Examination Center, Affiliated Hospital of Inner Mongolia Medical University, Hohhot, Inner Mongolia, China

**Keywords:** biomarker, bone metastasis, cross-cancer comparison, exosome, miRNA, multi-omics, targeted therapy, tumor microenvironment

## Abstract

Bone metastasis is a highly prevalent complication in patients with advanced prostate, breast, and lung cancers, which significantly affects the patient’s prognosis. In recent years, the integration of multi-omics technologies has provided unprecedented insights into the systematic analysis of the highly heterogeneous tumor microenvironment at a systemic level. This review begins with a cross-cancer comparison, systematically outlining the functional similarities and differences in key microenvironment components (e.g., tumor-associated macrophages, cancer-associated fibroblasts, osteoclasts, and T cells) in bone metastasis across these three cancer types. It emphasizes how miRNAs mediate intercellular communication via exosomes to coordinately regulate immune evasion, stromal activation, and bone metabolic reprogramming. We further explored the translational potential of miRNA-based liquid biopsies (e.g., miR-141-3p and miR-34a) for diagnosing and prognosticating bone metastasis in breast, prostate, and lung cancers. Finally, this article looks ahead at how integrating multi-omics data with AI predictive models can overcome current delivery and safety challenges, advancing miRNA research towards the ultimate goal of precision medicine.

## 1 Introduction

Bone metastasis is one of the most common complications of advanced malignant tumors and significantly affects the quality of life and prognosis of patients. Prostate, breast, and lung cancers are the three most common cancers prone to bone metastasis. Studies have shown that over 70% of patients with metastatic breast cancer develop bone metastasis, making it the primary site of distant metastasis (1). Prostate cancer also exhibits a high affinity for bone, with approximately 1.5%-4.2% of patients presenting with bone metastasis at the initial diagnosis. During disease progression, this proportion can increase significantly to 85%-100% (2). Compared to the former, the incidence of bone metastasis in lung cancer is slightly lower but remains a common metastatic pattern. A large population-based study showed that approximately 34%-39% of deceased lung cancer patients develop bone metastasis during the course of their disease (3). Bone metastasis usually indicates that the disease has reached an advanced stage. Current treatments for bone metastasis primarily include bone-targeted agents (BTAs), radiotherapy, surgical intervention, systemic anti-tumor therapy, and pain management. However, none of these approaches have achieved satisfactory outcomes (4).

The tumor microenvironment (TME) (5) is composed of non-cancerous cells within the tumor (e.g., immune cells, fibroblasts, and endothelial cells), the extracellular matrix, and various signaling molecules secreted by these components. Continuous and complex interactions between tumor cells and the TME profoundly influence tumor initiation, progression, metastasis, and the response to therapy, playing a decisive role in this process (6).

Multi-omics, as a novel technology, provides an effective approach to our understanding of the TME. The integration of radiomics, pathomics, genomics, and transcriptomics has significant research implications and value for the comprehensive evaluation of the TME and tumor prognosis assessment (7). Traditional single-omics approaches (e.g., transcriptomics and proteomics) can only partially reveal the regulatory network of miRNAs, with issues of false positives and negatives. Multi-omics integrative analyses (including genomics, transcriptomics, proteomics, and metabolomics) provide a more comprehensive and accurate depiction of miRNA interaction networks. This strategy has significantly advanced research on cancer mechanisms, biomarker discovery, and the development of personalized therapies (8).

miRNAs are a subclass of endogenous ncRNAs, typically approximately 22 nucleotides in length, expressed in multicellular organisms (9).

miRNAs regulate the expression of specific genes through post-transcriptional mechanisms, thereby participating in fine-tuning various physiological processes. Currently, approximately 2,500 miRNAs have been identified in the human genome, and it is estimated that their regulatory scope covers more than half of protein-coding genes. miRNAs primarily achieve broad and precise regulation of the gene expression by complementary pairing with the 3′-untranslated region (3′-UTR) of their target mRNAs, subsequently inducing mRNA degradation or inhibiting translation (10). MicroRNAs (miRNAs) are crucial gene expression regulators and play a significant role in the occurrence, progression, and outcome of tumors. Substantial evidence indicates that specific miRNAs are involved in various stages of bone metastasis, further highlighting their critical roles in the pathogenesis of bone metastatic diseases (11).

Current research has revealed the role of some miRNAs in the TME (5) of bone metastasis in single cancer types (e.g., breast, prostate, and lung cancers) (12–14); however, significant gaps remain in the systematic analysis of cross-cancer regulatory networks from a multi-omics perspective, hindering medical progress. This review systematically elaborates on the characteristics of the TME (5) revealed by multi-omics, the core regulatory role of miRNAs, and the prospects for clinical translation, all from a cross-cancer comparative perspective.

### 1.1 The regulatory role of the TME in cancer bone metastasis

**Figure 1:**
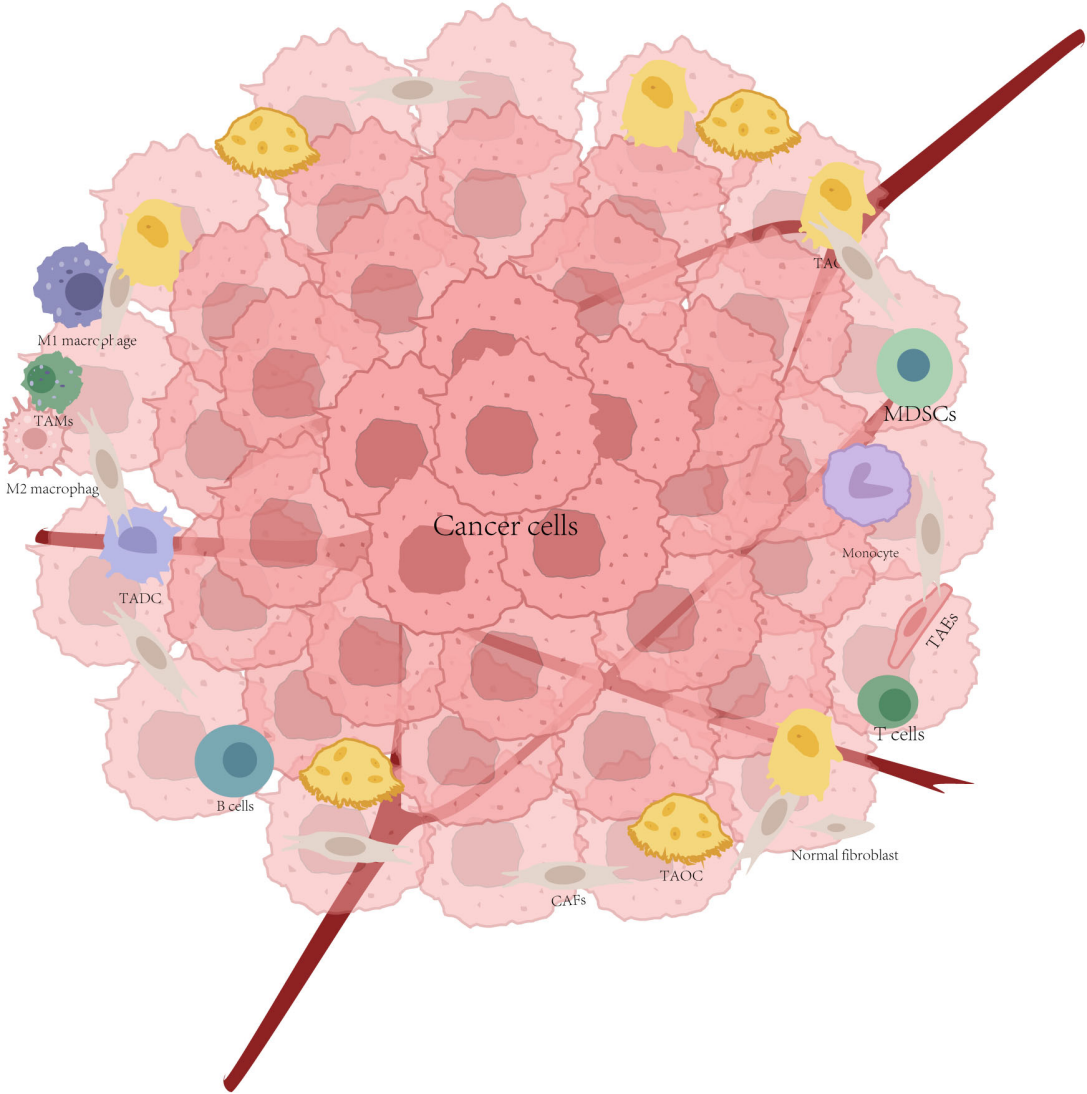
Cells in the tumor microenvironment.

Bone metastasis of cancer begins when tumor cells detach from the primary site, disseminate through the circulatory system (circulating tumor cells, CTCs), and colonize the bone (becoming disseminated tumor cells [DTCs]). The bone marrow microenvironment provides a unique niche for DTC dormancy; however, the molecular mechanisms underlying the initiation, maintenance, and reactivation of this dormant state remain unclear. Recent evidence reveals that the dormant state of DTCs is reversible, with its “on” and “off” switches bidirectionally regulated by bone marrow stromal cells, particularly osteoblasts and osteoclasts. These two cell types precisely control the dormancy and proliferation of DTCs through direct interactions or paracrine factors (15). After colonizing the bone marrow, CTCs remain dormant in the vascular and endosteal niches, thereby evading clearance and long-term survival. However, these cells can be reactivated after years of quiescence, subsequently driving pathological bone destruction through proliferation and alterations in the osteocyte function (16). The TME (5) is a complex ecosystem composed of tumor cells, stromal cells (e.g., CAFs, TECs, CAAs, and MSCs), and immune cells (e.g., T cells, macrophages, and NK cells). Traditional therapies primarily target tumor cells, but in recent years, there has been a shift towards targeting the cellular network within the TME, particularly with immune checkpoint inhibitors and cell therapies (e.g., CAR-T). Stromal and immune cells form a dynamic network in the TME, which can promote tumor progression and activate anti-tumor immunity (17), as shown in **Figure 1**.

### 1.2 Advantages of multi-omics technology in studying the TME

Rapid advances in biotechnology have led to a series of innovative omics technologies, enabling researchers to systematically acquire multi-level molecular information, such as genomes, epigenomes, transcriptomes, proteomes, and metabolomes. These technologies encompass both bulk omics and single-cell omics approaches, allowing for the precise characterization of different molecular layers at previously unattainable scales and resolutions. This provides an unprecedented holistic perspective for deciphering tumor heterogeneity, microenvironmental interactions, and drug resistance mechanisms (18). Genomics aims to decipher the genetic information encoded in DNA sequences, which governs cellular structure and function by regulating gene expression levels. This serves as the cornerstone for understanding how genomic alterations lead to human diseases (19, 20). As a direct manifestation of genomic activity, dynamic changes in the transcriptome decode the cellular reprogramming process under physiological and pathological conditions. Through a transcriptome analysis, researchers can uncover alterations in gene expression profiles, thereby elucidating their pivotal roles in the pathogenesis of cancer. RNA sequencing technology (RNA-seq) and spatial transcriptomics have significantly deepened our understanding of tumors (21, 22). Tumors exhibit a significant degree of inter- and intratumoral heterogeneity, often manifesting as phenotypically and functionally distinct cell populations (23). However, bulk omics techniques obscure the important characteristics of different cell subpopulations, providing only average measurements across multiple cells (24). The advent of single-cell technology has provided a powerful solution for studying omics features at a single-cell resolution.

## 2 Differences in TME cell composition among various bone metastases:

### 2.1 Breast cancer

In the TME (5) of breast cancer bone metastasis, malignant epithelial cells are significantly more abundant than other cell types. A reclustering analysis was performed on 13,865 malignant ECs from the primary tumor (PT) and lymph node metastasis (LM) samples, identifying four distinct subgroups: G0, G1, G2, and G. The G2 subgroup showed higher proportions in PT, LM, and other metastasis (OM) samples, while the G0 subgroup was most predominant in bone marrow (BM) samples. However, research by Li et al. demonstrated that the State 1 subpopulation manifests early in the trajectory in both PT and BoM samples, exhibiting high stemness characteristics. The mIHC results showed an increased proportion of pCAFs in late-stage samples, with pericytes and mCAFs being significantly more abundant in BM samples than in PT samples. Analysis of fibroblast proportions in the BoM revealed a notable increase in myofibroblasts and FAP+ inflammatory cells. Functional exploration indicated that myofibroblasts and FAP+ inflammatory cells in the BoM actively participate in processes such as cell proliferation, adhesion, complement, and coagulation cascades, while the overall proportion of immune cells decreases. Zhu et al. regrouped 18,504 T-NK cells and categorized them into T-memory cells, CD8+ T cells, Treg cells, NK cells, and CD4+ T cells based on marker gene expression levels. Among these, CD8+ T cells constituted the largest proportion of the BM samples. Li et al. observed a relative increase in cytotoxic NK-T cells and identified six TAM subpopulations (TAM 1-6), with all except TAM3 predominantly present in BoM, involved in cell adhesion, immune response, and immune regulation. Breast cancer bone metastasis is primarily characterized by naïve B-cells, although their overall proportion remains low (25, 26). The bone metastasis microenvironment of breast cancer exhibits a triple hallmark: malignant epithelial cell dominance, stromal remodeling (marked increase in pericytes/mCAFs/myofibroblasts), and immune suppression (overall immune cell reduction, but enrichment of specific CD8+ T cells and TAM subsets), as shown in **Table 1**.

**Table 1:** Breast cancer bone metastasis cell composition and characteristics.

Cell category	Cell subtypes	Key features/consensus
Malignant epithelial cells	G0 subgroup (dormant)	The proportion is significantly higher than that of the primary tumor (PT) and lymph node metastasis (LM), making it the dominant subgroup in bone metastasis.
	State 1 subgroup (high stemness, poor prognosis)	This high-dryness subgroup is enriched in bone metastases and serves as the initiating cells for metastasis.
Cancer-associated fibroblasts	pCAFs (proliferative CAFs)	The proportion is relatively high in bone metastases, and the high expression of its marker STMN1 is associated with advanced breast cancer and a poor prognosis.
	mCAFs (stromal CAFs)	The proportion in bone metastases is significantly higher than in primary lesions.
	Myofibroblasts & FAP+ CAFs	These two types of CAFs are significantly increased in bone metastasis and serve as key drivers of tissue remodeling.
immune cells	Total immune cells	Compared to PT and LM, the overall proportion of immune cells in bone metastases is lower, showing a trend towards an “immune desert.”
	CD8+ T cells	It is pointed out that it accounts for a considerable proportion in bone metastasis; while the cytotoxic NK-T cells are relatively increased, their function may be inhibited.
	Myeloid cells (e.g., TAMs)	Myeloid cells (particularly multiple subsets of TAMs) are significantly enriched in bone metastases, involved in immune suppression and cell adhesion.
	B cells	Mainly composed of naïve B cells, but the overall proportion is low.

### 2.2 Prostate cancer

Owing to the adoption of a rapid tissue dissociation protocol, the overall abundance of tumor cells is relatively low, averaging 3.4% (27). Feng et al. found that epithelial cells accounted for up to 35.40% of the TME (28). Feng et al.’s paper reports “35.4%” as the proportion of epithelial cells across all samples, reflecting their dominant role in the tumor microenvironment. In contrast, Kfoury et al.’s paper cites “3.4%” as the proportion of tumor cells within specific tumor tissue samples, indicating their relative scarcity in immune studies, which aligns with the study’s immune cell-prioritized sampling strategy. Therefore, the data from Feng et al.’s paper is more compelling. T/NK cells account for 35.66%. Although T/NK cells constitute a relatively large proportion, the CCL20-CCR6 signaling pathway may lead to T lymphocyte exhaustion. Although NK cells can be recruited to the general area of bone metastasis, they may not infiltrate the tumor. Macrophages constitute 8.31% of the population. Significant changes have been observed in tumor specimens, with notable populations of tumor-inflammatory monocytes and tumor-associated macrophages showing the high expression of monocytes and macrophages, respectively. TAM exhibits the characteristic expression pattern of M2 macrophages (29) and has been proven to suppress anti-tumor immune responses in a wide range of cancers (30). Endothelial cells are primarily present in the tumor stroma, accounting for approximately 5.85% of the cells. Based on transcriptomic differences, PC bone metastases can be classified into three subgroups: MetA, MetB, and MetC (31, 32). High endothelial cell density is associated with the MetB subtype and a poor prognosis. Fibroblasts account for 5.71% of the cell population. In MetB metastases, the density of smooth muscle actin (SMA+) and ERG+ stromal cells is significantly higher than that in MetA metastases, while the density of stromal cell-derived factor 1 (SDF1+) and decorin (DCN+) stromal cells is significantly lower in MetB metastases than in MetA metastases. The average bone density within metastatic lesions is 13%; however, there is considerable variation among patients, ranging from 0% to 50% of the metastatic volume. Although bone density is not correlated with MetA-C scores, it is positively correlated with PSA scores and negatively correlated with the prognosis (27, 28, 33). In summary, despite significant variations in tumor cell proportions (3.4% vs. 35.4%) due to methodological differences (e.g., tissue dissociation protocols) across studies, the bone metastasis microenvironment of prostate cancer consistently exhibited an immune infiltration profile dominated by T/NK cells (35.66%) and macrophages (8.31%). Within this environment, T cell exhaustion, M2-type TAM polarization, and endothelial-fibroblast matrix remodeling (MetB subtype) collectively contribute to an immunosuppressive microenvironment. Additionally, bone density heterogeneity is closely associated with the patient prognosis, as shown in **Table 2**.

**Table 2:** Prostate cancer bone metastasis cell composition and characteristics.

Cell type	Proportion occupied	Key features/consensus
Epithelial cells	35.40%	There are significant differences. Kfoury et al. used a rapid tissue dissociation protocol, resulting in a very low proportion. The study by Feng et al. may reflect more comprehensive cell capture or metastases at different stages.
T/NK cells	35.66%	T cells are the main infiltrating immune cells. Difference: The quantitative methods vary (cell counting vs. tissue volume density), leading to significant disparities in proportions. Kfoury et al. emphasize the functional state of T cells (exhaustion).
Fibroblasts	5.71%	Consensus: Fibroblasts are a crucial component of the stroma. Bergström et al. conducted the most comprehensive characterization of them and discovered that specific molecules (e.g., high POSTN, low DCN) are associated with the prognosis.
B cells	4.88%	Partial consensus. Kfoury et al. observed that B-cell depletion is a systemic feature; spatiotemporal data show that B cells account for a certain proportion, possibly reflecting heterogeneity among patients or sampling sites.
Macrophages	8.31%	Consensus: Macrophages are the main component of the myeloid lineage. Kfoury et al. further revealed their immunosuppressive subtypes and the function of CCL20 overexpression.
Endothelial cells	5.85%	Consensus: Endothelial cells are a key component of the TME. Bergström et al. clearly linked their high density to the aggressive subtype (MetB) and a poor prognosis.
Mast cells	1.58%	Participate in the regulation of allergic reactions and inflammation

### 2.3 Lung cancer

Tumor cells accounted for the highest proportion of bone metastases, with a significant increase in the proportion of senescent cells. The senescence-positive signature was significantly associated with bone metastasis characteristics and poor OS in patients. In lung cancer bone metastases, T cells had the second highest proportion, but CTLs were exhausted. In the tumor-infiltrated bone microenvironment, senescence of CD4Tstr cells, accompanied by the involvement of a pro-inflammatory secretory phenotype, may promote angiogenesis to support tumor colonization. B cells account for a moderate proportion of bone metastases, with PLCG2+ B cells being the most prevalent. PLCG2+ B cells play a crucial role in the early activation of immune responses (34, 35). The proportion of myeloid cells was moderate, with a gradual decline in bone marrow cell function, including a reduced immunosuppressive capacity and weakened anti-inflammatory properties. However, CCL3 myeloid cells are dominant in metastatic bone tumors. M2-related genes showed a sustained upregulation trend, indicating that the TME reprograms myeloid cells toward enhanced immunosuppressive activity.

The proportion of fibroblasts is moderate, with DCN+ CAFs prevalent in bone metastases. The proportion of endothelial cells is relatively low, and studies have shown that bone marrow-derived vascular endothelial cells exhibit significant cellular senescence. This senescent state exacerbates the endothelial-mesenchymal transition (EndMT) process and promotes pathological angiogenesis. Among them, the transcription factor SOX18 was identified as a key molecule in this process, and its overexpression is closely associated with a poor response to immunotherapy and an adverse prognosis in patients with non-small cell lung cancer. A higher proportion of mast cells may be involved in inflammation and immune regulation at bone metastasis sites (36, 37). In lung cancer bone metastases, senescence and functional remodeling of tumor cells and various immune cells (particularly CD4 Tstr cells, myeloid cells, and endothelial cells) collectively establish an immunosuppressive and pro-angiogenic microenvironment, which significantly facilitates tumor colonization and is associated with a poor patient prognosis, as shown in **Table 3**. Currently, the mechanisms underlying the preference of lung cancer bone metastasis for “osteolytic” or “osteoblastic” types remain unclear. Future research should integrate single-cell multi-omics, spatial transcriptomics, and organoid models to systematically elucidate their cellular interaction networks and molecular regulatory pathways.

**Table 3:** Composition and characteristics of bone metastasis cells in lung cancer.

Cell type	Findings of Liu et al.	Findings of Wang et al.	Consensus and core characteristics
tumor cells	Highest proportion, enriched AZGP1+ subtype	The proportion of senescent cells significantly increased, with higher CNV and highly metastatic characteristics.	Bone metastasis is dominated by tumor cells with higher malignancy and a senescent phenotype.
T cells	The proportion is the second highest, with CTL exhaustion	CD4Tstr (senescent stress) enrichment, reduced naïve T cells	T cell infiltration but dysfunctional: manifested as exhaustion, senescence, and imbalance in subtype proportions, collectively leading to immune suppression.
B cells	Moderate proportion, PLCG2+ B cells dominate	Proportion lower than the primary lesion	B cells are present but may have altered functionality, with an overall low level of infiltration.
myeloid cells	Moderate proportion, CCL3+ myeloid cells dominate	Not emphasizing the proportion	Myeloid cells polarize towards pro-tumor (e.g., M2-like) and pro-inflammatory (CCL3+) phenotypes.
CAFs/fibroblasts	Moderate proportion, dominated by DCN+ CAFs	The proportion is higher than that of the primary lesion.	Fibroblasts are active and participate in constructing a fibrotic, immunosuppressive stromal microenvironment.
Endothelial cells	Proportion is relatively low	Vascular endothelial cells (VasECs) undergo senescence and endothelial-mesenchymal transition (EndMT), lymphatic endothelial cells (LECs) decrease, and SOX18 is highly expressed.	Dysfunction of the vascular system promotes tumor survival and metastasis, while reducing lymphatic vessels.
Mast cell	proportionally high	Not mentioned	May be involved in the inflammation and immune regulation of bone metastases

## 3 Differences in TME of bone metastases

### 3.1 Similarities

#### 3.1.1 Tumor cells dominate with high malignancy

Breast cancer: significant increase in epithelial cells (tumors) and the presence of highly stem-like and metastatic subpopulations (e.g., state 1/G0).

Prostate cancer: Epithelial cells accounted for a high proportion of some data (e.g., 35.4% in spatiotemporal data), exhibiting high heterogeneity.

Lung cancer: The proportion of tumor cells in bone metastases is the highest, exhibiting malignant characteristics, such as senescence and high CNV.

#### 3.1.2 T cells are the main immune component, but they are dysfunctional

Breast cancer: The proportion of CD8+ T cells varies, but the overall T-cell function is suppressed.

Prostate cancer: T/NK cells are the primary immune infiltrating cells but exhibit an exhausted phenotype.

Lung cancer: The proportion of T cells is the second highest but manifests as exhaustion (CTL), senescence (CD4Tstr), and a reduction in naïve T cells.

#### 3.1.3 Macrophages/TAMs enrichment promotes immunosuppression

Breast cancer: TAMs are significantly enriched in bone metastases and are involved in immunosuppression and cell adhesion.

Prostate cancer: Macrophages are the main myeloid components of an immunosuppressive subtype (TAM).

Lung cancer: polarization of myeloid cells towards pro-tumor (M2-like) and pro-inflammatory (CCL3+) phenotypes.

#### 3.1.4 CAFs are active, driving stromal remodeling

Breast cancer: pCAFs (proliferative type) and mCAFs (stromal type) are significantly increased, driving tissue remodeling.

Prostate cancer: Fibroblasts are a key component of the stroma, with high POSTN and low DCN being associated with a poor prognosis.

Lung cancer: DCN+ CAFs dominate, constructing a fibrotic and immunosuppressive microenvironment.

#### 3.1.5 Reduced B-cell infiltration and weakened immune function

The proportion of B cells was low in all three cancer types, suggesting impaired adaptive immune responses.

#### 3.1.6 Endothelial cell dysfunction promotes tumor progression

Prostate cancer: endothelial cell density associated with aggressive subtypes and a poor prognosis.

Lung cancer: Endothelial cells undergo senescence and EndMT, with the high expression of SOX18 and reduced lymphatic vessels.

### 3.2 Differences

#### 3.2.1 Characteristics of tumor cells and metastasis types

Breast cancer: The existence of dormant subpopulations (G0) and highly stem-like subpopulations (State 1), with the latter driving metastasis.

Prostate cancer: epithelial cells are highly heterogeneous and encompass multiple subtypes that are associated with osteoblastic or osteolytic metastases.

Lung cancer is characterized by an aging phenotype and high CNV, predominantly metastasizing to the bones.

#### 3.2.2 Characteristics of the immune microenvironment

Breast cancer: overall presents an “immune desert” state with a low proportion of total immune cells, but a relative increase in cytotoxic NK-T cells.

Prostate cancer: a high proportion of T cells but severe exhaustion, systemic reduction of B cells, and well-defined functions of macrophage subtypes.

Lung cancer: concurrent T-cell senescence and exhaustion and enrichment of CD4 T str cells with a pro-angiogenic phenotype.

#### 3.2.3 CAF subtypes and functions

Breast cancer: both pCAFs and mCAFs showed significant increases in driving proliferation and ECM remodeling, respectively.

Prostate cancer: CAFs express markers, such as POSTN, which are closely related to the prognosis.

Lung cancer: predominantly DCN+ CAFs, leaning towards immunosuppression.

#### 3.2.4 Involvement of bone tissue components

Breast cancer: osteoblasts are not individually annotated, but CAFs and myeloid cells dominate bone remodeling.

Prostate cancer: bone tissue composition is clearly defined, including osteoblasts and osteoclasts, with a high bone density and significant heterogeneity.

Lung cancer: Bone tissue components are not emphasized, with a greater focus on the myeloid and T-cell-regulated bone microenvironment.

#### 3.2.5 Research methods and proportional differences

Breast cancer: multiple studies strongly agree that the cell state is directly linked to the clinical prognosis.

Prostate cancer: significant variations in cell proportions across studies (e.g., epithelial cells ranging from 3.4% to 35.4%) reflect the influence of sampling and experimental methodologies.

Lung Cancer: focusing on cellular state transitions (e.g., senescence, EndMT) rather than mere proportions.

#### 3.2.6 Conclusion

The bone metastasis TMEs of the three types of cancer exhibited high consistency in terms of tumor dominance, immune suppression, CAF activation, B-cell reduction, and vascular abnormalities, reflecting the common immune evasion and stromal remodeling mechanisms of the bone metastatic microenvironment. However, each type of cancer exhibits unique cellular state preferences and functional architectures: breast cancer favors stemness/dormancy transitions and immune deserts; prostate cancer emphasizes bone tissue interactions and T-cell exhaustion; lung cancer features aging and T-cell dysfunction. These similarities and differences provide crucial insights for understanding the mechanisms of cancer-specific bone metastasis and developing targeted therapies (e.g., anti-aging, anti-CAFs, and immune activation strategies).

## 4 Regulation of key cells in TME by miRNA

### 4.1 Breast cancer

In breast cancer bone metastasis, miRNAs orchestrate a complex network of intercellular communication that sustains tumor growth, immune evasion, and osteolytic destruction.

#### 4.1.1 Tumor-associated macrophages

Tumor-associated macrophages (TAMs) are a crucial immune cell population in the TME that exhibit significant functional and phenotypic heterogeneity (38). TAMs exhibit significant phenotypic plasticity, polarizing into an M2-like phenotype with pro-tumor and immunosuppressive functions through co-evolution with tumor cells (39, 40). Among the various cell types present in the TME, tumor-associated macrophages serve as critical regulatory hubs for interactions between tumors and the immune system. Recent advancements in single-cell sequencing technology, combined with a growing body of research, have revealed the functional diversity and heterogeneity of TAMs as well as the mechanisms underlying their interactions within the TME. This suggests that TAMs could serve as innovative therapeutic targets for cancer treatment, thereby facilitating the development of personalized anti-cancer strategies (41). Studies have shown that miR-19a-5p can target the proto-oncogene Fra-1. This study regulated the expression of its downstream signaling molecules (including STAT3, pSTAT3, and VEGF) under both *in vitro* and *in vivo* conditions, thereby inhibiting the induction of M2 macrophage polarization (42, 43). A study found that the miR-23a/27a/24–2 gene cluster can regulate macrophage polarization, thereby promoting the progression of breast cancer. Additionally, research suggests a dual feedback loop that facilitates tumor progression, with the miR-23a/27a/24–2 gene cluster acting as a hub. This loop functions by mediating the dynamic switch of TAMs between pro-inflammatory (M1) and anti-inflammatory (M2) phenotypes (44).

Tumor cell-derived exosomal miR-148b-3p can target TSC2 to regulate macrophage polarization through the TSC2/mTORC1 signaling pathway, thereby promoting breast cancer cell proliferation and potentially affecting their migration and invasion capabilities (45). Zhou et al. suggested that tumor cell-derived exosomes deliver miR-184-3p to macrophages, targeting and inhibiting EGR1 while downregulating the JNK signaling pathway, thereby inducing macrophage polarization towards the M2 phenotype and ultimately promoting tumor progression (46). Zhou et al. found that miR-382 might inhibit the progression and metastasis of breast cancer by targeting PGC-1α and regulating macrophage metabolism to weaken M2 polarization (47). Research by Lian et al. revealed that hypoxia triggers a cascade reaction promoting breast cancer invasion by downregulating miR-143-3p in exosomes derived from hypoxic breast cancer cells: this miRNA inhibits RICTOR, thereby blocking the polarization of M2-type macrophages (48). Studies have confirmed that miR-223 is overexpressed in IL-4-activated macrophages and can shuttle into breast cancer cells via exosomes. Once inside cancer cells, it enhances their invasive ability by regulating the Mef2c-β-catenin signaling pathway, thereby exerting its oncogenic function (49). Further research has revealed that CAFs can polarize monocytes into anti-inflammatory M2-type macrophages, and this pro-tumor effect partially depends on the delivery of monocyte-derived miR-181a to breast cancer cells via extracellular vesicles, thereby regulating their PTEN/Akt signaling axis (50).

#### 4.1.2 Cancer-associated fibroblasts

Beyond TAMs, cancer-associated fibroblasts (CAFs) are educated by tumor-derived miRNAs to become key accomplices in metastatic progression. By secreting various chemokines, they serve as a hub and driving force that promotes tumor epithelial-mesenchymal transition (EMT), angiogenesis, drug resistance, and invasion/metastasis (51–53).

The study revealed a specific phenomenon: Conditioned medium (54) derived from basal-like breast CAFs uniquely suppresses the estrogen receptor (ER) expression in breast cancer cells by overexpressing miR-221/miR-222, an effect not observed in CAFs from other sources (55). Studies have confirmed that miR-222 induces normal fibroblasts (NFs) to acquire a CAF phenotype by directly targeting the Lamin B receptor (56); the conditioned medium (54) secreted by these transformed CAFs was ultimately proven to significantly enhance the migration and invasion capabilities of breast cancer cells, thereby revealing the carcinogenic role of miR-222 (57). Research by Donnarumma et al. confirmed that CAF-derived exosomes enhance stemness, EMT, and anchorage-independent growth of breast cancer cells by delivering high levels of miR-21, -143, and -378e, thereby significantly increasing their invasive potential (58). Sheng et al. revealed that CAF-derived exosomal miR-92a promotes breast cancer cell migration and invasion by reducing G3BP2 and may represent a potential novel tumor marker for breast cancer (59). The study by Zhu et al. found that breast cancer cells deliver miR-425-5p through exosomes, driving the transformation of mammary fibroblasts into CAFs in a TGFβRII (TGFβ1) receptor-dependent manner, thereby promoting tumor growth and metastasis (60). Dongwei Dou et al. found that overexpression of miR-92 leads to increased nuclear translocation of YAP1, followed by upregulation of PD-L1. Transfection with miR-92 inhibitors enhanced LATS2 expression, which was observed to reduce YAP1 nuclear translocation and subsequently downregulate PD-L1. Cancer-associated fibroblast-derived exosomes inhibit immune cell function in breast cancer via the miR-92/programmed cell death receptor ligand 1 (PD-L1) pathway, and transfection of miR-92 increases the proliferation and migration of breast cancer cells (61). Exosomes derived from CAFs upregulate the expression of TXN through the highly expressed LINC01711 via the miR-4510/NELFE axis, thereby activating the glycolytic pathway and ultimately enhancing the proliferation, migration, and invasion abilities of breast cancer cells (62). In a breast cancer model, Wu et al. demonstrated that miR-16 and miR-148a were enriched in exosomes from FAK-deficient CAFs by constructing fibroblast-specific inducible FAK knockout (cKO) mice, which are key molecules mediating the reduction of tumor cell activity and metastatic potential (63). The research results of Cosentino et al. and others indicate that the miR-9/EFEMP1 (EGF-containing fibulin extracellular matrix protein 1 axis) is a key mechanism driving the transformation of normal fibroblasts (NFs) into CAF-like cells under the influence of triple-negative breast cancer signals (64). Studies by Chen et al. showed that CAF-derived miR-500a-5p promotes the proliferation and metastasis of cancer cells by targeting and inhibiting ubiquitin-specific peptidase 28 (USP28), high levels of miR-500a-5p are transferred into tumor cells and downregulate the expression of USP28, thereby promoting breast cancer cell proliferation, metastasis, and EMT (65). Ansari et al. revealed that miR-146b-5p is expressed at low levels in CAFs, and its downregulation is associated with malignant progression of breast cancer. Restoring the levels of this miRNA in the stroma can impair its ability to support EMT and metastasis in cancer cells, suggesting that it may be a potential target for stromal intervention (66). Studies have revealed that miR-200 synergistically drives the expression of fibronectin and LOX through both direct and indirect (via Fli-1/TCF12) pathways, with its mediated ECM remodeling being a critical step in promoting breast cancer invasion and metastasis (67).

#### 4.1.3 Myeloid-derived suppressor cells

Myeloid-derived suppressor cells (MDSCs) are a group of immature myeloid cells that proliferate under various pathological conditions. As key components of the tumor immune microenvironment, they play a central immunosuppressive role by negatively regulating the immune function (68, 69). MDSCs are divided into three subsets: monocyte-derived MDSCs (M-MDSCs), polymorphonuclear (granulocytic) MDSCs, and early stage MDSCs (eMDSCs) (70). Research by Kim et al. revealed that the loss of miR-155 in cancer cells upregulates its target C/EBP-β, which in turn alters the cytokine profile to recruit more myeloid-derived suppressor cells (MDSCs), ultimately reshaping the immune microenvironment and promoting tumor growth and invasion (71). Similarly, in a bone marrow-specific SOCS3 knockout mouse model, Zhang et al. demonstrated that SOCS3 deficiency activates the Wnt/mTOR pathway through the miR-155/C/EBPβ axis, inhibiting autophagy and differentiation of early myeloid-derived suppressor cells, thereby shaping an immunosuppressive microenvironment and promoting tumor progression (72). Deng et al. found that DOX chemotherapy activates a positive feedback loop between MDSCs and IL-13+ Th2 cells, where MDSC-derived exosomal miR-126 drives Th2 responses, whereas Th2-derived IL-13 further promotes MDSC activation and miR-126 release, collectively exacerbating chemotherapy resistance and metastasis in breast cancer (73).

#### 4.1.4 Dendritic cells

Dendritic cells (DCs) are antigen-presenting cells that capture, process, and present antigens to lymphocytes to initiate and regulate adaptive immune responses. Although DCs function by mediating anti-tumor responses, cancer cells secrete factors that inhibit DC differentiation and their potential to activate immune responses (74, 75). Studies have reported that DCs interact with and alter the TME by releasing microRNAs (miRNAs) (12, 14). In dendritic cells, the upregulation of miR-155 not only enhances their migration, antigen uptake, and maturation (manifested by the increased expression of CD80 and MHC II) but also further promotes T cell proliferation and the secretion of effector factors IFN-γ and IL-2 (76). A study by Liang et al. identified miR-22 as a key negative regulator of the DC function, which impairs antitumor immunity by targeting the p38 signaling pathway and suppressing the IL-6/Th17 axis. Targeting and inhibiting miR-22 is a viable strategy for enhancing the efficacy of DC immunotherapy (77). The study identified miR-5119, which is downregulated in tumor-associated DCs, as it directly targets key immunosuppressive molecules, such as PD-L1 and IDO2, thereby reshaping the immune microenvironment to reverse T-cell exhaustion and enhance anti-tumor immunity (78).

#### 4.1.5 Tumor-associated endothelial cells

Tumor-associated endothelial cells (TAEs), as key components of the TME, construct a physical and functional barrier that restricts the infiltration of immune cells. Their unique metabolic reprogramming ability actively suppresses anti-tumor immune responses by competitively depleting nutrients and other means (79). The study confirmed through northern blotting and RT-qPCR that miR-126 and its host gene EGFL7 are specifically highly expressed in human umbilical vein endothelial cells, while their expression is downregulated in human breast tumor tissues. This miRNA directly targets VEGFA and PIK3R2, and its overexpression effectively inhibits the activity of the VEGF/PI3K/AKT signaling pathway in breast cancer cells (80).

#### 4.1.6 T cells

Tregs are an important subset of T lymphocytes that play a key role in regulating immune responses and have the potential to suppress inflammatory reactions (81). In breast cancer, Tregs not only serve as the central hub of immunosuppression but also drive tumor progression through dual immune-dependent and -independent functions. Their subtype-specific prognostic value and multidimensional mechanisms of action provide new directions for targeted therapies (82). Soheilifar et al. found that miR-182 promotes immunosuppression by constructing a network that inhibits T-cell activation signals (FOXO1/NFAT/IL-2), thereby driving lineage differentiation of T cells into Tregs (83). Research by Pei et al. revealed that the lncRNA SNHG1 upregulates the expression of IDO by adsorbing miR-448, thereby promoting the differentiation of regulatory T cells and playing a key role in breast cancer immune escape (84). A study by Hu et al. showed that miR-21 drives the expansion of CCR6+ regulatory T cells in the TME by targeting the PTEN/Akt signaling pathway. Silencing miR-21 can reshape the tumor immune microenvironment and enhance anti-tumor immunity, providing a novel targeted strategy for breast cancer immunotherapy (85).

#### 4.1.7 Tumor-associated osteoblasts

Hsu et al. found that mammary tumor-associated osteoblasts promote breast cancer EMT and metastasis by secreting CXCL5 to activate the ERK/MSK1/Elk-1 signaling axis, which drives the upregulation Snail (86). Circulating miR-218 drives breast cancer bone metastasis through a dual mechanism: directly inhibiting osteoblast collagen synthesis, while simultaneously blocking bone matrix maturation via the inhibin βA/Timp3 axis, collectively disrupting bone homeostasis (87).

#### 4.1.8 Tumor-associated osteoclasts

Breast cancer bone metastasis is characterized by osteolytic lesions, with the core mechanism being the tumor-induced excessive activation of osteoclasts. Pathological bone resorption driven by osteoclasts not only causes bone damage, but also provides a favorable microenvironment for tumor cell colonization and growth, thereby creating a vicious cycle (88).

Cai et al. discovered that the loss of the miR-124 expression in bone tissue promotes osteoclast formation by upregulating IL-11, thereby accelerating the process of bone metastasis in breast cancer. Cancer cell-derived miR-124 inhibits the survival and differentiation of osteoclast precursor cells and its expression level is significantly correlated with the patient prognosis (89). During breast cancer bone metastasis, miR-16 accelerates bone destruction by promoting osteoclastogenesis, while miR-133a and miR-223 exert inhibitory effects. These three miRNAs form a crucial molecular network that regulates the balance of the bone microenvironment (90). Wu et al. found that ER+ breast cancer cells create an osteoclast-enriched pre-metastatic bone microenvironment by co-secreting IBSP (Integrin-Binding Sialoprotein) and exosomal miR-19a. Chlorogenic acid can inhibit this process by blocking IBSP, thus offering a new strategy for preventing and treating bone metastasis (91).

Research by Yuan et al. revealed that breast cancer cells deliver miR-21 to osteoclasts via exosomes, promoting osteoclast differentiation and formation of a pre-metastatic bone microenvironment through PDCD4 regulation. Serum levels of miR-21 may serve as a potential diagnostic marker for bone metastasis in breast cancer (92). A study by Kim et al. showed that RAS activation in breast cancer cells stimulates exosome-mediated delivery of pro-osteoclastogenic miRNAs, such as miR-494-3p. This miRNA promotes osteoclast formation and suppresses osteogenic differentiation through a bidirectional regulatory mechanism by targeting LGR4 (leucine-rich repeat-containing G-protein-coupled receptor 4) and Sema3A, thereby inducing osteolytic bone metastasis (93). The study by Liu et al. showed that breast cancer cells deliver lncRNA-MIR193BHG via exosomes, acting as a competitive endogenous RNA (ceRNA) to competitively bind miR-489-3p and relieve its inhibitory effect on DNMT3A, thereby driving osteoclast differentiation and osteolysis. This signaling axis provides a novel targeted strategy for treating bone metastasis in breast cancer (94, 95).

**Figure 2:**
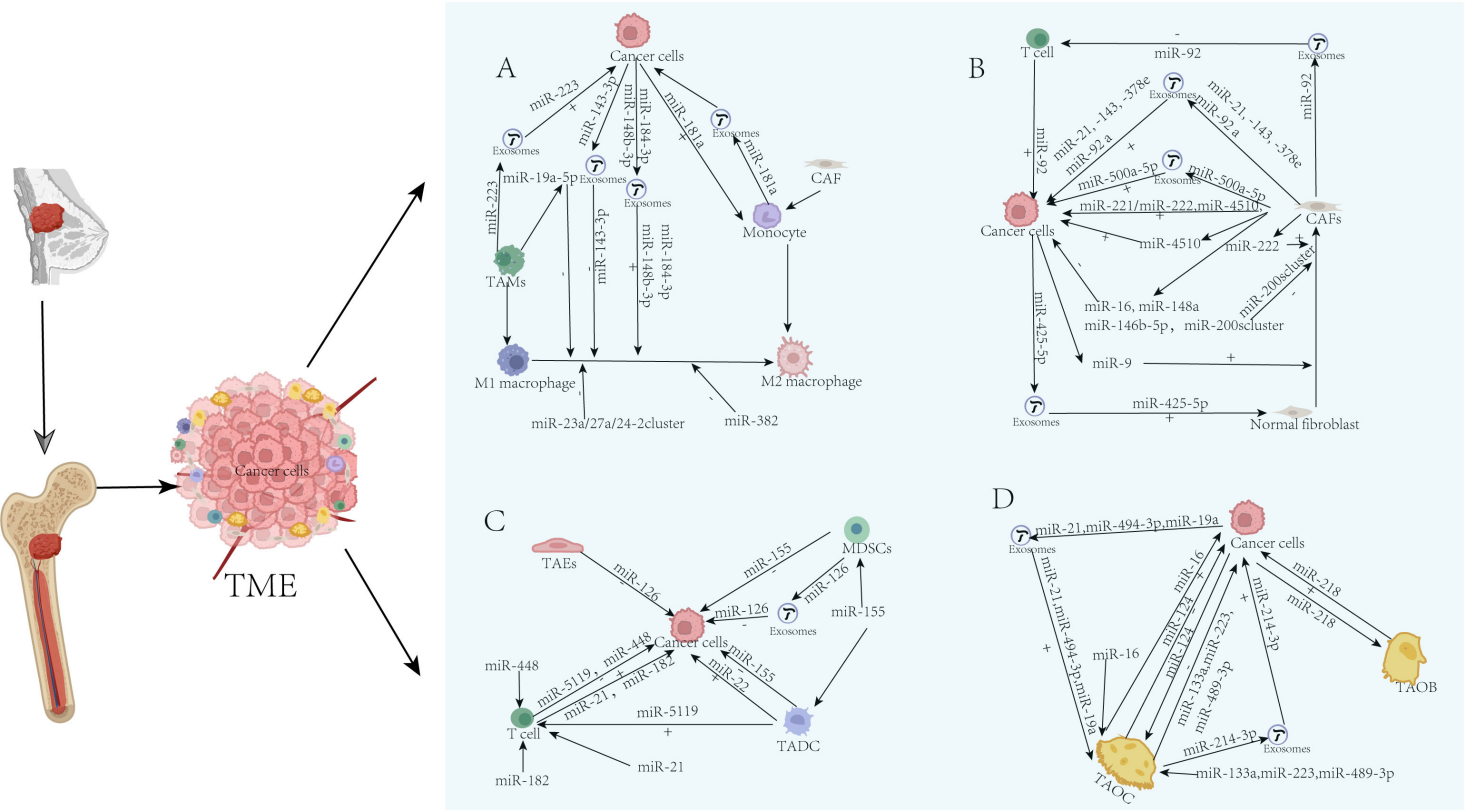
**(A)** TME-resident cell-secreted miRNAs and their role in regulating BC progression and metastasis. The bone TME includes many types of cells, including Tumor-associated macrophages (TAMs ), Cancer-associated fibroblasts (CAFs), Myeloid-derived suppressor cells (MDSCs),Tumor-associated dendritic cells(TADCs), Tumor-associated endothelial cells (TAEs), T cells, Tumor-associated osteoblasts(TAOB) and Tumor-associated osteoclasts(TAOCs), which secrete miRNAs and regulate BC invasion, homing, and progression in the bone. **(A)** TAMs. **(B)** CAFs. **(C)** TAEs, TADCs, T cells, and MDSCs. **(D)** TAOB, TAOCs. “+” indicates promotion; “-”indicates inhibition.

These miRNAs promote or inhibit breast cancer progression through various mechanisms, as shown in **Table 4**, **Figure 2**.

**Table 4:** Breast cancer bone metastasis miRNA regulates key cells in the TME.

miRNA	Source cells	Target gene/function	Impact on bone metastasis of breast cancer
miR-19a-5p	Tumor-associated macrophages	Targeting Fra-1 to inhibit M2 polarization	Inhibiting tumors: overexpression can reduce the invasion and migration of BC cells
miR-23a/27a/24-2	Tumor-associated macrophages	Regulating macrophage polarization	Inhibiting tumors: its downregulation promotes M2 polarization, driving tumor progression
miR-148b-3p	Tumor cell exosomes	Regulating macrophage polarization	Promoting tumors: stimulates the proliferation of breast cancer cells and may affect their migration and invasion capabilities
miR-184-3p	Tumor cell exosomes	Regulating macrophage polarization	Promoting tumor progression: targeting EGR1 to inhibit the JNK signaling pathway induces macrophage polarization towards the M2 phenotype and synergistically promotes tumor progression.
miR-382		Targeting PGC-1α and regulating macrophages	Inhibiting tumors: regulates macrophage metabolism to weaken their M2 polarization, thereby inhibiting the progression and metastasis of breast cancer.
miR-181a	Monocyte exosomes	Delivered to breast cancer cells via extracellular vesicles, thereby regulating the PTEN/Akt signaling axis to achieve	Promoting tumor growth: CAFs can polarize monocytes into anti-inflammatory M2 macrophages
miR-143-3p	Tumor cell exosomes	Regulating macrophage polarization	Inhibiting tumors: targets RICTOR to block the polarization of M2-type macrophages
miR-223	IL-4 activated macrophage exosomes	Through the Mef2c-β-catenin pathway	Promoting tumors: enhances the invasive ability of BC cells
miR-221/miR-222	Cancer-associated fibroblasts	Suppressing ER expression	Promoting tumors: confers aggressiveness to BC cells
miR-222	Cancer-associated fibroblasts	Targeting Lamin B Receptor	Promoting tumors: induces normal fibroblasts to acquire CAF characteristics, promoting BC migration and invasion
miR-21, -143, -378e	Cancer-associated fibroblast exosomes	–	Promoting tumors: enhance stem cell properties, EMT, and anchorage-independent growth of BC cells
miR-92 a	Cancer-associated fibroblast exosomes	Reducing G3 BP 2	Promoting tumors: plays a promoting role in the migration and invasion of breast cancer cells
miR-425-5p	Tumor cell exosomes	TGFβRII (TGFβ1) receptor-dependent manner	Promoting tumors: driving the transformation of breast fibroblasts into CAFs
miR-92	Cancer-associated fibroblast exosomes	Targeting LATS2 enables YAP1 to bind to the enhancer region of PD-L1	Promoting tumors: induces apoptosis and damage in T cells
miR-4510	Cancer-associated fibroblasts	Highly expressed LINC01711 upregulates the expression of TXN via the miR-4510/NELFE axis	Promoting tumor growth: activates the glycolytic pathway, ultimately enhancing the proliferation, migration, and invasion capabilities of breast cancer cells.
miR-16, miR-148a	Cancer-associated fibroblasts	FAK-deficient CAFs exhibit enrichment of miR-16 and miR-148a in their exosomes.	Inhibiting tumors: reduces the activity and metastatic ability of tumor cells
miR-9	tumor cells	Targeting EFEMP 1	Promoting tumors: drives the transformation of breast fibroblasts into CAFs
miR-500a-5p	Cancer-associated fibroblast exosomes	Targeted inhibition of ubiquitin-specific peptidase 28 (USP28)	Promoting tumors: promotes the proliferation and metastasis of cancer cells
miR-146b-5p	Cancer-associated fibroblasts	–	Inhibiting tumors: Suppressing the pre-EMT and metastatic effects of BC cells
miR-200s family	Cancer-associated fibroblasts	–	Inhibiting tumors: prevents the transformation of normal fibroblasts into CAFs and reducing BC invasion and migration
miR-155		Targeting C/EBP-β	Inhibiting tumors: in the absence of SOCS3, it upregulates its target C/EBP-β, recruiting more myeloid-derived suppressor cells (MDSCs) to inhibit MDSC autophagy and differentiation in a bone marrow-specific SOCS3 knockout mouse model.
miR-126	Myeloid-derived suppressor cell exosomes	Activation of IL-13+ Th2 cells	Promoting tumors: forms a positive feedback loop, mediating chemotherapy resistance and metastasis
miR-22	Dendritic cells	Targeting p38, downregulating IL-6	Promoting tumor growth: inhibits the anti-tumor effects of dendritic cells
miR-155		Promoting the proliferation of T cells and the secretion of effector factors IFN-γ and IL-2	Inhibiting tumors: enhances their migration, antigen uptake, and maturation (manifested as the increased expression of CD80 and MHCII)
miR-5119	Dendritic cells	Targeting PD-L1	Inhibiting tumors: suppresses T cell exhaustion and restoring the CD8+ T cell function
miR-126	Tumor-associated endothelial cells	Targeting VEGFA, PIK3R2	Inhibiting tumors: overexpression can reduce the activity of the VEGF/PI3K/AKT signaling pathway
miR-182		Suppressing the network of T-cell activation signals (FOXO1/NFAT/IL-2)	Promoting tumors: drives the lineage differentiation of T cells into Tregs, thereby promoting immunosuppression
miR-448	CD4+ tumor-infiltrating lymphocytes	Targeting IDO	Inhibiting tumors: suppresses Treg cell differentiation and blocking BC immune escape
miR-21		Targeting PTEN	Promoting tumors: enhances Treg proliferation through the PI3K/Akt pathway
miR-218	Tumor cells	Inhibiting collagen synthesis in osteoblasts	Promoting tumors: inhibits osteoblast collagen synthesis and blocking bone matrix maturation through the inhibin βA/Timp3 axis
miR-124	Tumor cells	The deficiency of the miR-124 expression promotes osteoclastogenesis by upregulating IL-11.	Inhibiting tumors: miR-124 derived from cancer cells suppresses the survival and differentiation of osteoclast precursor cells
miR-16		Promoting the expression of osteolytic factors (RANKL, IL-1β, IL-6, PTHrP, and TNF)	Promoting tumors: promotes osteoclastogenesis and accelerate bone destruction
miR-133a,miR-223		Inhibiting the expression of osteolytic factors (RANKL, IL-1β, IL-6, PTHrP, and TNF)	Inhibiting tumors: suppresses osteoclast formation
miR-19a	Exosomes from ER+ breast cancer cells	Exosomal miR-19a delivered to osteoclasts to induce osteoclastogenesis	Promoting tumors: constructs an osteoclast-enriched pre-metastatic bone microenvironment
miR-21	Tumor cell exosomes	miR-21 promotes osteoclastogenesis by regulating PDCD4 protein levels	Promoting tumor growth: accelerates osteoclast formation and bone destruction
miR-489-3p		Inhibiting DNMT3A	Inhibiting tumors: suppresses osteoclast differentiation
miR-214-3p	Tumor-associated osteoclast exosomes	Targeting TRAF3	Promoting tumors: promotes osteoclast formation and osteolytic bone metastasis
miR-494-3p	RAS-activated tumor cells	By targeting LGR4 and Sema3A	Promoting tumors: stimulates osteoclast formation, inhibits osteogenic differentiation, thereby inducing osteolytic bone metastasis.

### 4.2 Prostate cancer

Unlike breast cancer, prostate cancer bone metastasis is characterized by a distinct miRNA profile that favors osteoblastic progression, as discussed below.

#### 4.2.1 Tumor-associated macrophages

Macrophages in the TME are divided into two main phenotypes, M1 and M2. M1 macrophages are characterized by pro-inflammatory activity and effective antigen presentation, while M2 macrophages exhibit anti-inflammatory properties, characterized by impaired antigen presentation, and are associated with immune tolerance (42). Research by Guan et al. revealed a novel mechanism by which tumor-associated macrophages promote the malignant progression of prostate cancer through exosome-mediated delivery of miR-95. This miRNA drives tumor proliferation and invasion by directly targeting JunB, and its expression level is significantly correlated with a poor patient prognosis, providing a new target for developing personalized therapeutic strategies targeting TAM tumor cell communication (96). Zhang et al. found that urine exosome-derived miR-203 exhibits dual potential as a novel diagnostic marker and therapeutic strategy by inducing M1 macrophage polarization and directly inhibiting the malignant behaviors of prostate cancer cells (97).

Rong et al. proposed that let-7b-5p enhances STAT1/3/5 phosphorylation by targeting SOCS1, thereby suppressing macrophage phagocytic function and promoting prostate cancer cell proliferation. Inhibition of this miRNA can reverse the immunosuppressive effect (98).

#### 4.2.2 Cancer-associated fibroblasts

A study by Liu et al. revealed that CAFS in the hypoxic TME deliver miR-500a-3p via exosomes, which target and suppress FBXW7 to activate the HSF1 signaling pathway, thereby driving the metastatic progression of prostate cancer. Both RT-qPCR and western blot analyses showed a significant decrease in FBXW7 expression after treatment with hypoxic exosomes (99). Research by Matsuda et al. has shown that in the androgen-sensitive prostate cancer microenvironment, CAF-derived miR-3121-3p exerts a tumor-suppressive effect by targeting NKX3–1 and inhibiting the dedifferentiation process of cells. The common hypoxic characteristics of the TME may further disrupt this finely regulated mechanism (100). Shan discovered that cancer-associated fibroblasts deliver miR-423-5p via exosomes, which suppresses the expression of GREM2 through the TGF-β pathway, thereby driving chemotherapy resistance to paclitaxel in prostate cancer. In this study, exosome treatment increased the expression of TGF-β mRNA and protein in PC cells, while the reduction of exosomal miR-423-5p led to an increase in the expression of TGF-β mRNA and protein in PC cells (101). Tumor-associated endothelial cells

This study elucidated that miR-323 promotes VEGF-A-mediated tumor angiogenesis by directly targeting the 3’UTR of AdipoR1 mRNA, revealing a novel mechanism by which it regulates the TME in prostate cancer (102).

#### 4.2.3 T cells

Tao et al. discovered that miR-195 and miR-16 reshape the immune microenvironment by suppressing the expression of PD-L1, enhancing T-cell activity, and synergizing with radiotherapy, collectively improving the treatment response and prognosis in patients with prostate cancer (103).

#### 4.2.4 Tumor-associated osteoblasts

Research by Zou et al. found that prostate cancer cells deliver miR-1275 via exosomes, activating osteoblast proliferation and differentiation through the SIRT2/RUNX2 signaling axis, providing a new mechanistic explanation for osteogenic lesions in the bone metastatic microenvironment of prostate cancer (104). Androgen receptor deficiency or inhibition upregulates exosomal circ-DHPS, which sequesters miR-214-3p via the ceRNA mechanism, thereby activating CCL5 (C-C chemokine ligand 5) secretion in osteoblasts to construct a chemotactic microenvironment that promotes prostate cancer bone metastasis (105). Prostate cancer cells deliver miR-26a-5p, miR-27a-3p, and miR-30e-5p via exosomes, synergistically inhibiting BMP-2-mediated osteogenic differentiation signaling, thereby driving the progression of osteosclerotic lesions characterized by impaired osteogenic activity in the bone microenvironment (106). Ye et al. proposed that prostate cancer cells deliver miR-141-3p via exosomes, which activates the p38MAPK signaling pathway by targeting and inhibiting DLC1, thereby driving osteoblast activity and the formation of an osteogenic bone metastasis microenvironment (107). Liu et al. found that overexpression of miR-140-3p (delivered via osteoblast-derived exosomes) significantly enhanced the phosphorylation of AKT at T308 and S473 sites, activating AKT and further promoting GSK3β phosphorylation and mTOR activation, thereby stimulating the proliferation, invasion, and migration capabilities of PCa cells (LNCaP). Inhibition of miR-140-3p (treated with a miR-140-3p inhibitor) significantly reduced the phosphorylation levels of AKT and mTOR, weakening the activity of this pathway. Liu et al. proposed that osteoblast-derived exosomal miR-140-3p activates the AKT/mTOR pathway by targeting ACER2 and inhibiting cellular autophagy, thereby driving the progression and metastasis of prostate cancer (108). Furesi et al. discovered that prostate cancer cells deliver miR-26a-5p, miR-27a-3p, and miR-30e-5p via exosomes, collectively inhibiting BMP-2-mediated osteogenic differentiation signaling. While promoting the proliferation of osteoprogenitor cells, they simultaneously block their mineralization capacity, ultimately leading to abnormal bone formation in osteosclerotic lesions (106).

#### 4.2.5 Tumor-associated osteoclasts

The study by Tamura et al. showed that prostate cancer can induce the production of pathological osteoclasts. These osteoclasts release extracellular vesicles rich in miR-5112 and miR-1963, which synergistically drive bone resorption and inhibit bone formation by targeting Parp1 in osteoclasts and Hoxa1 in osteoblasts. This process independently remodels the bone metastatic microenvironment of the RANKL pathway (109). Exosomes derived from prostate cancer cells (PC-3) synergistically downregulate miR-214 and inhibit the NF-κB signaling pathway, thereby suppressing osteoclast differentiation and the expression of specific markers, ultimately delaying the progression of bone metastasis (110). Research by Han et al. found that downregulation of miR-181b-5p during osteoclast differentiation inhibits the malignant progression of prostate cancer by targeting and suppressing Oncostatin M, thereby regulating the balance of the IL-6/AREG/OPG expression. This provides a theoretical basis for targeting this axis as a therapeutic approach to bone metastasis (111). Ma et al. discovered that prostate cancer cells deliver miR-152-3p to osteoclasts via small vesicles, targeting and suppressing the expression of the transcription factor MAFB, thereby driving osteoclast differentiation and the process of osteolytic bone metastasis; intervention with this miRNA can effectively delay bone structure destruction (112).

#### 4.2.6 Human mesenchymal stem cells

Liu et al. found that overexpression of miR-375 can activate the Wnt/β-catenin pathway (upregulating TCF-1, LEF-1, and β-catenin, while downregulating Cyclin D1 and Axin2), thereby promoting osteogenic differentiation of mesenchymal stem cells. Conversely, inhibition of miR-375 reduces the activity of this pathway and suppresses osteogenic differentiation. This study discovered that prostate cancer cells deliver miR-375 via exosomes, which target and suppress DIP2C to activate the Wnt/β-catenin signaling pathway, thereby driving osteoblastic bone metastasis and tumor progression (113). Cancer cells deliver miR-940 via exosomes, targeting ARHGAP1 and FAM134A to promote osteogenic differentiation of stem cells, revealing that specific miRNAs can serve as key determinants in defining the bone metastasis phenotype (osteogenic/osteolytic) (114). Cheng et al. proposed that prostate cancer cells deliver lncRNA NEAT1 via exosomes, which competitively bind to miR-205-5p through the SFPQ/PTBP2 axis, thereby upregulating the expression of RUNX2 and ultimately driving the osteogenic differentiation process of bone marrow mesenchymal stem cells, thus providing a potential target for the treatment of prostate cancer bone metastasis (115).

**Figure 3:**
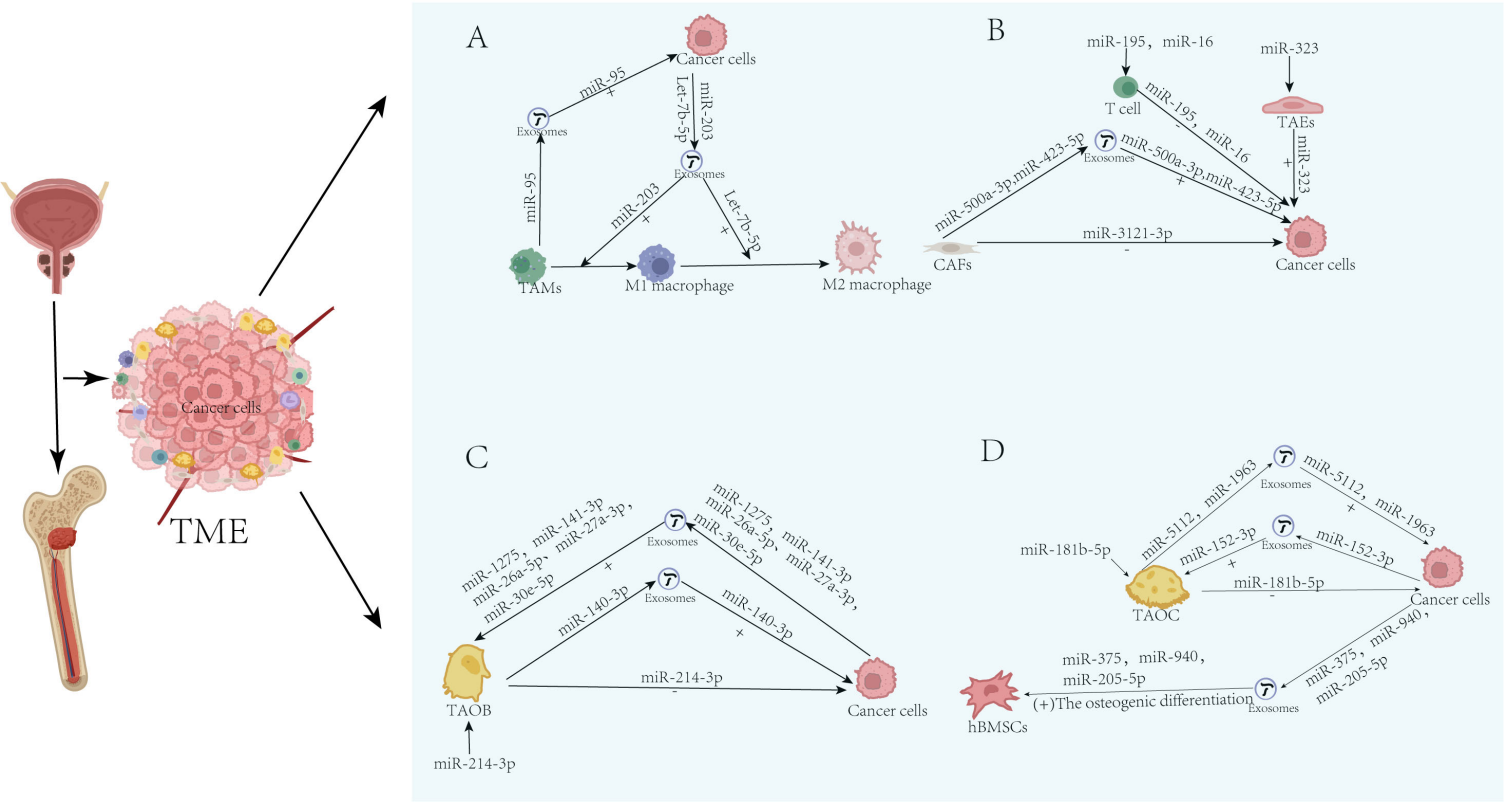
TME-resident cell-secreted miRNAs and their role in regulating PC progression and metastasis. **(A)** TAMs. **(B)** CAFs. T cells, TAEs. **(C)** TAOB. **(D)** TAOC. “+” indicates promotion; “-” indicates inhibition.

These miRNAs promote or inhibit prostate cancer progression through various mechanisms, as shown in **Table 5**, **Figure 3**.

**Table 5:** Bone metastasis of prostate cancer miRNA regulates key cells in the TME.

MicroRNA	Source cells	Target gene/function	Impact on bone metastasis of prostate cancer
miR-95	Tumor-associated macrophage exosomes	Directly targeting JunB drives tumor proliferation and invasion	Promoting tumors: promotes tumor proliferation and invasion
miR-203	Cancer cells	Inducing M1 polarization of macrophages	Inhibiting tumors: suppresses malignant behaviors of prostate cancer cells
Let-7b-5p	PC-3 cell exosomes	SOCS1	Promoting tumor growth: induces M2 polarization via the SOCS1/STAT pathway to facilitate tumor progression
miR-3121-3p	CAFs	NKX3-1	Inhibiting tumors: suppresses tumor dedifferentiation and prevents bone metastasis
miR-500a-3p	CAFs (hypoxic) exosomes	FBXW7	Promoting tumors: facilitates metastasis through the FBXW7/HSF1 axis
miR-423-5p	CAFs exosomes	GREM2	Promoting tumors: enhances chemotherapy resistance and promoting metastasis through the TGF-β pathway
miR-323		Directly targeting the 3’UTR of AdipoR1 mRNA promotes VEGF-A-mediated tumor angiogenesis	Promoting tumors: promotes tumor angiogenesis
miR-195, miR-16		PD-L1	Inhibiting tumors: enhances T-cell activity and synergizing with radiotherapy
miR-1275	Prostate cancer cell exosomes	SIRT2	Promoting tumors: promotes osteoblast proliferation and bone-forming metastasis through SIRT2/RUNX2
miR-26a-5p, miR-27a-3p, miR-30e-5p	Prostate cancer cell exosomes	Inhibition of BMP-2-mediated osteogenic differentiation signaling	Promoting tumors: these microRNAs play a key role in the inhibition of osteoblast activity mediated by prostate cancer.
miR-214-3p		Activate osteoblast CCL5 secretion	Promoting tumors: constructs a chemotactic microenvironment that facilitates bone metastasis of prostate cancer
miR-141-3p	Prostate cancer cell exosomes	DLC1	Promoting tumors: downregulates DLC1 to facilitate the formation of a pre-osteogenic microenvironment
miR-140-3p	Osteoblast exosomes	Targeting ACER2	Promoting tumorigenesis: activates the AKT/mTOR pathway and inhibits cellular autophagy
miR-5112, miR-1963	Osteoclast exosomes	Targeting Parp1 in osteoclasts and Hoxa1 in osteoblasts respectively	Promoting tumors: synergistically driving bone resorption and inhibiting bone formation
miR-214	Prostate cancer cell exosomes	–	Inhibiting tumors: suppresses the NF-κB pathway, inhibits osteoclast differentiation, and reduces bone resorption
miR-152-3p	Prostate cancer cell exosomes	Targeting MAFB	Promoting tumors: targets osteoclasts to drive the progression of osteolytic bone metastasis
miR-181b-5p		Targeted inhibition of OSM	Inhibiting tumors: regulates the balance of the IL-6/AREG/OPG expression
miR-375	Prostate cancer cell exosomes	DIP2C	Promoting tumors: activates the Wnt pathway, promotes osteogenic differentiation and bone metastasis
miR-940	Prostate cancer cell exosomes	ARHGAP1, FAM134A	Promoting tumors: promotes osteogenic differentiation and enhances bone metastasis
miR-205-5p	Prostate cancer cell exosomes	RUNX2	Promoting tumors: promotes RUNX2-mediated osteogenic differentiation

Unlike the “osteolytic” characteristics of breast cancer, the miRNA regulatory network of prostate cancer bone metastasis exhibits a distinct “osteogenic programming” core. It activates key pathways like Wnt/β-catenin and RUNX2 through molecules like miR-375/940/1275, while simultaneously employing inhibitory regulation of osteoclasts and immune cells (e.g., miR-214, let-7b-5p), collectively constructing an immunosuppressive microenvironment conducive to osteogenic growth. This network not only explains its unique radiological manifestations (high-density osteoblastic lesions) but also points to new directions for combination therapy: for instance, targeting miR-375 to disrupt the “osteogenic niche,” or combining it with miR-203 mimics to activate anti-tumor immunity.

### 4.3 Lung cancer

The mechanism of bone metastasis in lung cancer is complex and highly heterogeneous. Its miRNA regulatory network primarily focuses on promoting the aggressiveness of primary tumors, inducing immunosuppression, and constructing a vascularized niche, while its direct impact on the balance of bone remodeling remains relatively unclear.

#### 4.3.1 Tumor-associated macrophages

Chen et al. discovered that tumor cells transmit PCAT6 via exosomes, inducing macrophage M2 polarization through the miR-326/KLF1 pathway. These polarized macrophages further promote lung cancer cell metastasis by remodeling the TME, thereby forming a vicious tumor-immune cycle (116). Wei et al. discovered that M2 macrophages deliver miR-942 via exosomes, which targets and suppresses the expression of FOXO1, thereby activating the β-catenin signaling pathway, subsequently promoting the invasion, migration, and angiogenesis processes of lung adenocarcinoma cells (117). Studies by Arora et al. revealed that miR-34a-5p disrupts the positive feedback loop between KLF4 and IL-1β/miR-34a-5p by targeting KLF4, thereby polarizing macrophages from the M2 to the M1 phenotype. This subsequently inhibits the progression of non-small cell lung cancer by enhancing nitric oxide-mediated apoptosis (118). Overexpression of miR-103a induces macrophage polarization toward the M2 phenotype and upregulates the expression of IL-10, CCL18, and VEGF-A by targeting PTEN inhibition, thereby activating the PI3K/AKT and STAT3 signaling pathways. This mechanism was also validated in exosomes derived from hypoxic lung cancer cells and could be blocked by PI3K or STAT3 inhibitors, indicating the critical role of miR-103a in shaping an immunosuppressive tumor microenvironment (119). miR-1207-5p inhibits the STAT3/AKT signaling pathway by targeting CSF1, thereby blocking macrophage M2 polarization and tumor angiogenesis, ultimately suppressing lung cancer progression, and improving the prognosis (120).

#### 4.3.2 Cancer-associated fibroblasts

Zhang et al. found that downregulation of the miR-101 expression in CAFs promotes the proliferation, migration, and invasion abilities of lung cancer cells by releasing the targeted inhibition of CXCL12 and activating TME signaling (121). Lee et al. proposed that the high expression of miR-196a in CAFs promotes the migration and invasion abilities of lung cancer cells by targeting and inhibiting ANXA1, thereby releasing its regulation of CCL2 and driving the malignant progression of tumors (122). Exosomal LINC01833 derived from CAFs, the LINC01833/miR-335-5p/VAPA axis, was revealed to be the central pathway through which CAFs regulate the tumor immune microenvironment and drive the progression of NSCLC via exosome-mediated dual effects (promoting tumor progression and inducing M2 polarization) (123). Sun et al. discovered that CAFs deliver miR-3124-5p via exosomes, which drives the malignant progression of non-small cell lung cancer by negatively regulating TOLLIP, thereby relieving its suppression of the TLR4/MyD88/NF-κB signaling pathway (124).

#### 4.3.3 T cells

T cell exhaustion is a prominent feature in bone metastasis of lung cancer, and miRNAs play a crucial role in this process.

Zarogoulidis et al. discovered that targeting autophagy can break the dual barriers of immune suppression and chemotherapy resistance. Activation of the miR-155-dependent T-cell infiltration and apoptosis pathway provides a novel synergistic therapeutic strategy for advanced lung cancer (125).

Chen et al. discovered that ZEB1 transcriptionally represses miR-200, thereby relieving its targeting effect on PD-L1. This mechanism drives the functional exhaustion of CD8+ T cells and promotes tumor metastasis, revealing a non-autonomous regulatory link between epithelial and mesenchymal transition and immune suppression (126).

The study by Li et al. revealed that circRUNX1 enhances glycolysis and lactate production by adsorbing miR-145 to relieve its inhibition of HK2, thereby promoting regulatory T cell infiltration and immune escape, ultimately driving the progression of non-small cell lung cancer (127).

#### 4.3.4 Tumor-associated osteoclasts

Zhang et al. reported that 27-hydroxycholesterol inhibited the expression of miR-139, thereby relieving its targeted suppression of c-Fos. This subsequently activates the STAT3/c-Fos/NFATc1 signaling axis and enhances the synergistic binding between transcription factors (c-Fos→NFATc1, pSTAT3→Oscar), ultimately driving osteoclast differentiation in the microenvironment of lung adenocarcinoma (128).

**Figure 4:**
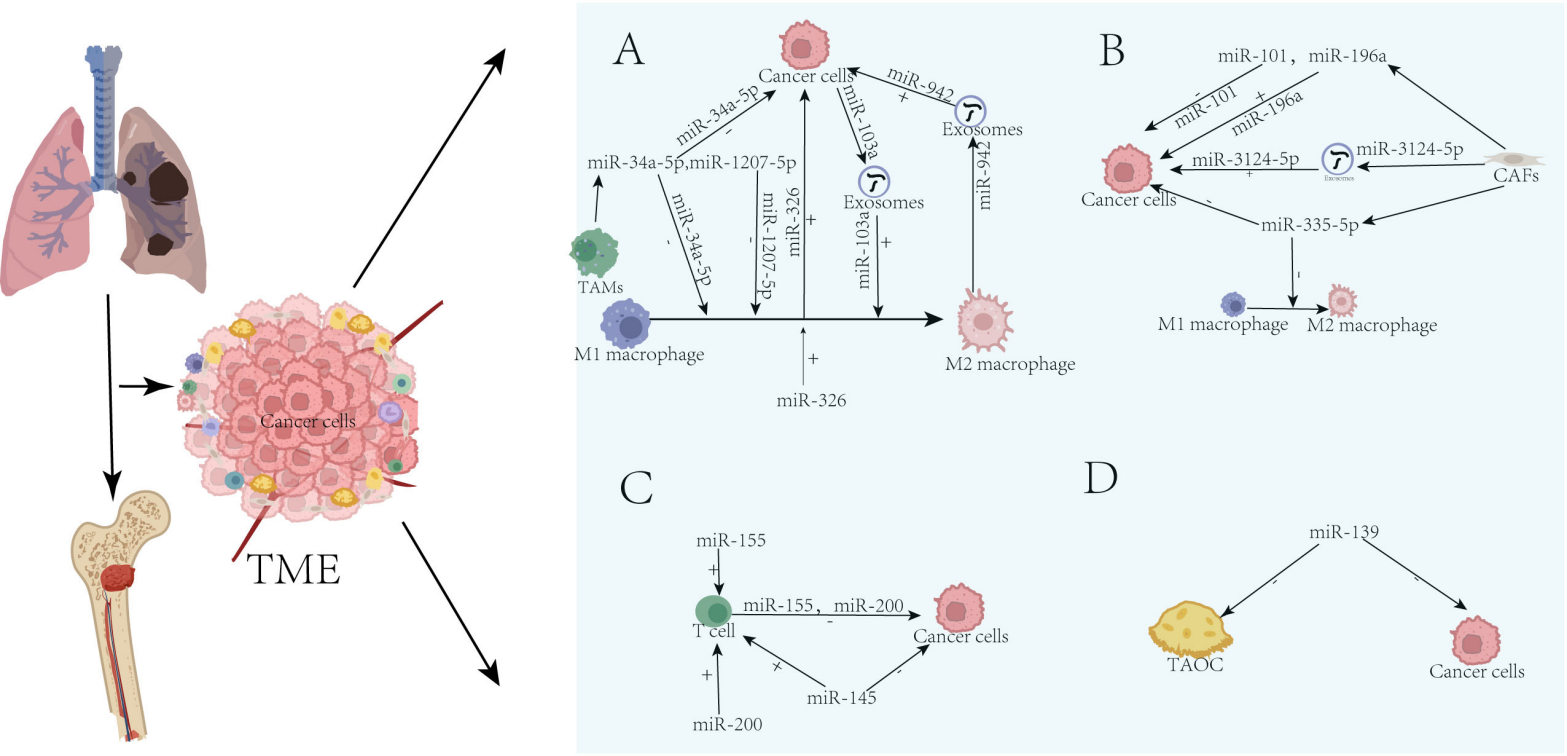
TME-resident cell-secreted miRNAs and their role in regulating lung cancer progression and metastasis. **(A)** TAMs. **(B)** CAFs. **(C)** T cells. **(D)** TAOC. “+” indicates promotion; “-”indicates inhibition.

These miRNAs promote or inhibit lung cancer progression through various mechanisms, as shown in **Table 6**, **Figure 4**.The miRNA regulatory network in lung cancer bone metastasis exhibits prominent microenvironmental characteristics. Its core mechanism lies in utilizing molecules such as miR-103a, miR-942, and miR-200 to predominantly construct an immunosuppressive (M2-TAMs, Treg, T-cell exhaustion) and highly vascularized niche to support metastatic colonization, while the direct reprogramming effect on bone metabolism is relatively secondary and ambiguous. This mechanistic emphasis is closely related to the high heterogeneity of lung cancer itself and the frequent occurrence of bone metastasis in advanced disease stages. Future therapeutic strategies should focus on disrupting this immunosuppression-angiogenesis axis (e.g., targeting miR-103a) and combining immune checkpoint inhibitors, which may prove more effective than directly intervening in bone metabolism.

**Table 6:** Bone metastasis of lung cancer miRNA regulates key cells in the TME.

MicroRNA	Source cells	Target genes and functions	Impact on bone metastasis
miR-326		PCAT6 induces macrophage M2 polarization through the miR-326/KLF1 pathway	Promoting tumors: reshapes the tumor microenvironment to further facilitate metastasis of lung cancer cells
miR-942	Tumor-associated macrophage exosomes	Targeted inhibition of FOXO1 to activate the β-catenin signaling pathway	Promoting tumors: facilitates the invasion, migration, and angiogenesis of lung adenocarcinoma cells
miR-34a-5p	Tumor-associated macrophages	Targeting KLF4 to disrupt its positive feedback loop with IL-1β/miR-34a-5p	Inhibiting tumors: suppresses the progression of non-small cell lung cancer
miR-1207-5p	Tumor-associated macrophages	Targeting CSF1, downregulating STAT3 and AKT signaling, reducing M2 characteristics, increasing M1 characteristics, and inhibiting lung cancer growth and metastasis.	Inhibiting tumors: suppresses the growth and metastasis of lung cancer
miR-103a	Tumor cell exosomes	Targeting PTEN, activating AKT and STAT3, promoting M2 polarization, and increasing the expression of pro-angiogenic factors.	Promoting tumor growth: drives macrophage polarization towards the M2 phenotype and form a tumor-promoting feedback loop
miR-101		Targeting CXCL12	Inhibiting tumors: blocks the ability of tumor cells to proliferate, form spheres, migrate, and invade, while increasing apoptosis.
miR-196a		Targeted inhibition of ANXA1	Promoting tumors: enhances the migration and invasion abilities of lung cancer cells
miR-335-5p	Cancer-associated fibroblasts	The LINC01833/miR-335-5p/VAPA axis is revealed as the core pathway through which CAFs regulate the tumor immune microenvironment.	Inhibiting tumors: suppresses tumor growth and metastasis
miR-3124-5p	Cancer-associated fibroblast exosomes	Negative regulation of TOLLIP relieves its inhibition of the TLR4/MyD88/NF-κB signaling pathway	Promoting tumors: drives the malignant progression of non-small cell lung cancer
miR-155		No direct target gene (N/A), autophagy inhibition combined with carboplatin treatment increases the expression of miRNA-155 and promotes TIL infiltration	Inhibiting tumors: may suppress metastasis and restore chemosensitivity.
miR-200	CD8+ tumor-infiltrating lymphocytes (TILs)	Targeting PD-L1, increasing CD8+ T cell infiltration, and reversing the exhausted CD8+ T cell phenotype	Inhibiting tumors: reduces tumor burden and metastasis
miR-145		Inhibiting HK2 to reduce glycolysis and lactate production	Inhibiting tumors: suppresses tumor growth and metastasis
miR-139		Targeted inhibition of c-Fos	Inhibiting tumors: suppresses osteoclast differentiation

### 4.4 Differences in MiRNA among different cancer types

#### 4.4.1 Similarities

##### 4.4.1.1 The core cellular messengers are highly consistent with their sources

The miRNA regulatory networks in the three types of cancer are highly dependent on the following three core cell types and their exosomes.

Tumor cells and their exosomes: This is the most direct source of microRNA, used to mediate intercellular communication and influence the distant microenvironment.

Tumor-associated macrophages (TAMs): as a “regulatory switch” in the TME, their polarization states (M1 anti-tumor/M2 pro-tumor) are precisely regulated by various microRNAs, playing a pivotal role in all three types of cancers.

CAFs and their exosomes: as the core of the tumor stroma, they are “educated” to strongly support tumor growth, invasion, and drug resistance by secreting factors such as microRNAs.

##### 4.4.1.2 Common biological processes and molecular pathways

Although the cancer types vary, the downstream biological processes regulated by miRNAs exhibit a high degree of conservation.

##### 4.4.1.3 Fine regulation of macrophage polarization

Promotes M2 polarization (pro-tumor): prostate cancer (Let-7b-5p), lung cancer (miR-103a, miR-326), and breast cancer (miR-184-3p).

Promotes M1 polarization or inhibits M2 polarization (anti-tumor) in prostate cancer (miR-203), lung cancer (miR-1207-5p), and breast cancer (miR-19a-5p, miR-143-3p).

###### 4.4.1.3.1 Targeted regulation of immune checkpoints

All three types of cancers have evolved mechanisms to enhance anti-tumor immunity by targeting PD-L1 through microRNAs: prostate cancer (miR-195/16), lung cancer (miR-200), and breast cancer (miR-5119).

Intervention targeting the osteoclast/osteoblast balance in bone metastasis

All three factors affect bone homeostasis through microRNAs, which is the common pathological basis of bone metastasis. Inhibiting osteoclasts (anti-tumor): Prostate cancer (miR-214), lung cancer (miR-139), and breast cancer (miR-489-3p).

###### 4.4.1.3.2 Promoting osteoclasts (pro-tumor) in breast cancer (miR-214-3p, miR-21, and miR-19a): shared key oncogenic signaling pathways

AKT/mTOR pathway: frequently mentioned in prostate cancer (miR-140-3p), lung cancer (miR-103a), and breast cancer (miR-181a), it primarily promotes cell survival and proliferation.

###### 4.4.1.3.3 STAT3 signaling: the core pathway related to miR-103a, miR-1207-5, and macrophage polarization in lung cancer

Wnt/β-catenin pathway: activated in prostate cancer (miR-375) and lung cancer (miR-942) to promote tumor progression.

##### 4.4.1.4 Shared key microRNA molecules

Some microRNAs play the same or similar roles in different types of cancer.

miR-214 promotes bone metastasis in both prostate cancer (miR-214-3p) and breast cancer (miR-214-3p), although the specific mechanisms differ slightly.

#### 4.4.2 Differences

##### 4.4.2.1 Cancer type-specific core mechanisms and microRNA

Breast cancer: constructing an Extremely Complex Immune and CAF

Regulatory network

Immune cell diversity: involved dendritic cells (miR-5119 targets PD-L1), myeloid-derived suppressor cells (miR-126 mediates chemotherapy resistance), and T lymphocytes (miR-448 inhibits Treg differentiation), with regulatory levels far exceeding those in the other two cancers.

CAF functional diversity: CAF-derived miRNAs cover a wide range of functions, including metabolic reprogramming (miR-4510 activates glycolysis), immune suppression (miR-92 promotes T-cell apoptosis via the YAP/PD-L1 axis), and stem cell properties (miR-21, -143, -378e cluster).

Prostate cancer: driving “osteogenic” bone metastasis.

miR-375, miR-940, and miR-205-5p directly promote osteogenic differentiation and bone formation, constructing osteogenic metastatic foci.

miR-3121-3p specifically inhibits tumor differentiation and maintains the malignant phenotype of tumors.

Lung Cancer: focusing on primary tumor invasion and angiogenesis.

miR-942: potently promotes angiogenesis by activating the β-catenin pathway.

miR-196a: targets ANXA1 specifically enhances the migration and invasion capabilities of lung cancer cells.

##### 4.4.2.2 Preference for pathological types of bone metastases

The three types of cancer exhibit distinctly different tendencies in regulating the bone microenvironment.

###### 4.4.2.2.1 Prostate cancer: a clear “osteogenic” preference

Its microRNA network is significantly biased towards activating osteoblasts and promoting new bone formation. This often results in prostate cancer bone metastases appearing as “high-density” osteoblastic destruction on imaging.

###### 4.4.2.2.2 Breast cancer: a distinct “osteolytic” preference

After removing dormancy-related miRNAs, their tendency to drive osteoclast formation and promote bone resorption and destruction is the most prominent feature. Multiple miRNAs (e.g., miR-214-3p, miR-19a, miR-21) work together to construct an efficient “bone-dissolving machine”.

###### 4.4.2.2.3 Lung cancer: tends to be “osteolytic” but with relatively vague mechanistic descriptions

These data indicate that it inhibits osteoclast differentiation through miR-139, suggesting its potential role in bone resorption. However, overall, the exploration and description of the specific mechanisms of bone metastasis (osteogenic/osteolytic) are not as in-depth and clear as the previous two.

##### 4.4.2.3 Contextual differences in microRNA functional orientation

The same miRNA may be “endowed” with different functions in the microenvironment of different cancers.

###### 4.4.2.3.1 miR-214-3p

In prostate cancer, it builds a chemotactic microenvironment by activating osteoblasts to secrete CCL5, thereby promoting tumor growth.

In breast cancer, it promotes osteoclastogenesis by targeting TRAF3 and thereby facilitating bone resorption.

Conclusion: Although both promote bone metastasis, they act on different cells in the skeletal system (osteoblasts vs. osteoclasts), reflecting “context specificity”.

###### 4.4.2.3.2 miR-223 (in breast cancer)

After the removal of bone marrow-derived miR-223, its pro-tumorigenic function, originating from IL-4-activated macrophages and promoting invasion, becomes dominant. This creates a potential divergence from its possible role (if any) in prostate and lung cancer.

#### 4.4.3 Conclusion

We have integrated the regulatory networks of key miRNAs in bone metastases of breast, prostate, and lung cancers, highlighting both common mechanisms across cancer types (such as the tumor-suppressive function of miR-34a and the pro-tumorigenic role of miR-21) and cancer-specific patterns (such as miR-375-mediated osteoblastic metastasis in prostate cancer and miR-942-driven angiogenesis in lung cancer), as shown in **Table 7**.

**Table 7:** Cross-cancer comparison of miRNA-mediated regulation in bone metastasis.

miRNA	Key cell type	Core pathway/mechanism	Role in breast cancer	Role in prostate cancer	Role in lung cancer	Common vs. distinct features
miR-214-3p	Osteoclasts/Osteoblasts	TRAF3 (BC); CCL5 activation (PC)	Promotes osteolysis	Promotes osteogenesis & chemotaxis	Not reported	Context-specific: osteoclast vs. osteoblast targeting
miR-34a	Tumor cells, Macrophages	TGF-β, p53, Notch, macrophage polarization	Tumor suppressor, in clinical trials	Under study	Tumor suppressor, in clinical trials	Shared tumor suppressor, under clinical development
miR-21	CAFs, Osteoclasts	PTEN/Akt, PDCD4, Treg expansion	Promotes CAF activity & osteolysis	Less emphasized	Associated with fibrosis & progression	Pro-tumor in BC & LC, stroma-focused in BC
miR-200 family	Tumor cells, T cells	PD-L1, EMT, T-cell exhaustion	Suppresses EMT	Not prominent	Reverses T-cell exhaustion	Immune modulation in LC, EMT suppression in BC
miR-155	DCs, MDSCs, T cells	C/EBP-β, autophagy, T-cell activation	Regulates MDSC recruitment	Not emphasized	Enhances T-cell infiltration	Immune regulator in BC & LC, cell type-dependent
miR-103a	Macrophages	PTEN/AKT/STAT3, M2 polarization	Not reported	Not reported	Drives M2 polarization & angiogenesis	LC-specific pro-tumor loop
miR-375	MSCs, Osteoblasts	Wnt/β-catenin, osteogenic differentiation	Not reported	Promotes osteogenic metastasis	Not reported	PC-specific osteogenic driver
miR-942	Macrophages	FOXO1/β-catenin, angiogenesis	Not reported	Not reported	Promotes angiogenesis & invasion	LC-specific angiogenic miRNA
miR-203	Macrophages, Tumor cells	M1 polarization, tumor suppression	Not reported	Induces M1 polarization	Not reported	PC-specific immunostimulatory
miR-489-3p	Osteoclasts	DNMT3A inhibition	Suppresses osteoclast differentiation	Not reported	Not reported	BC-specific anti-osteolytic

Overall, miRNAs play a critical role in bone metastasis of breast, prostate, and lung cancers, sharing some common mechanisms through the regulation of immune cells, bone cells, and signaling pathways; however, each cancer exhibits a unique miRNA expression profile and mode of action. These similarities and differences reveal the cancer type-specific molecular mechanisms and provide insights for the development of targeted therapies. For instance, therapies targeting shared pathways (e.g., PD-L1) may be effective across cancers, but require consideration of miRNA context specificity to avoid side effects. Future research should focus on crosstalk between miRNA networks across cancers to optimize personalized treatment strategies.

### 4.5 Potential difference mechanisms and biological basis

Although breast cancer, prostate cancer, and lung cancer share microenvironmental features such as immune suppression and stromal remodeling in bone metastasis, their bone metastasis phenotypes and miRNA regulatory networks exhibit significant differences. These differences may stem from the following aspects:

#### 4.5.1 Origin and molecular phenotype of tumor cells

Breast cancer (especially the triple-negative subtype) tends to secrete osteoclast-activating factors (RANKL and PTHrP) and promotes osteoclastogenesis through miR-21 and miR-214-3p, leading to “osteolytic” lesions.

Prostate cancer often relies on androgen receptor signaling, with its cells tending to secrete osteogenic factors (BMP-2, RUNX2 and WNT ligands) that promote osteoblast activation, thereby forming “osteogenic” metastatic lesions. This study also found that miR-375 and miR-940 directly promote osteogenic differentiation by activating the Wnt/β-catenin pathway.

The mechanism of bone metastasis in lung cancer is relatively complex, possibly related to high tumor heterogeneity and diverse pathological subtypes. The regulatory role of its miRNA network in the osteogenic/osteoclastic balance remains unclear and warrants further exploration.

#### 4.5.2 Heterogeneity and cellular composition of the bone microenvironment

In breast cancer bone metastasis, CAFs (especially mCAFs and FAP+ CAFs) are significantly increased, potentially promoting osteolytic destruction through the secretion of matrix-degrading enzymes and chemokines.

Higher endothelial cell density and fibrotic matrix expression of POSTN were observed in prostate cancer bone metastases, which are associated with the MetB subtype and poor prognosis, suggesting that a vascular-rich microenvironment may be more conducive to osteogenic metastasis.

The proportion of senescent cells increases in bone metastasis of lung cancer, and the senescence-associated secretory phenotype may support tumor colonization by promoting angiogenesis and immunosuppression rather than directly regulating bone remodeling.

#### 4.5.3 Epigenetics and metabolic reprogramming

The expression of miRNAs is itself regulated by epigenetic mechanisms, and the metabolic state of different cancers may influence their exosomal miRNA profiles:

Hypoxic microenvironment can upregulate the expression of miR-500a-3p in CAFs (prostate cancer) or promote the release of miR-103a from lung cancer cell-derived exosomes, thereby regulating macrophage polarization.

Metabolites (such as lactic acid and cholesterol derivatives) may indirectly shape the bone metastasis phenotype by influencing miRNA stability or transcription factor activity.

## 5 Multi-omics integration and biomarker association

Multi-omics technologies are advancing cancer research from single-dimensional descriptions to multi-dimensional systems-level analyses. This big data-driven systems biology paradigm ultimately aims to build models capable of predicting cancer cell behavior and treatment responses, thereby achieving personalized medicine (129). scRNA-seq and spatial transcriptomics have confirmed the heterogeneous expression of miRNAs in the TME, which imposes higher demands on their delivery strategies: cell-specific targeting must be achieved to ensure that therapeutic miRNAs exert their intended effects at the correct locations (130). Single-cell and spatial transcriptomic technologies provide a critical biological foundation for constructing high-precision, reproducible diagnostic models by analyzing the cellular and spatial specificity of the miRNA expression within the TME (131). Chaturvedi et al. systematically identified 33 oncogenic and 17 tumor-suppressing key miRNAs (LCmiRs) in lung adenocarcinoma by integrating a WGCNA network analysis with multi-omics data. The study constructed their targeted regulatory networks, revealing that the loss of tumor-suppressing miRNAs leads to the activation of the cell cycle and TGF-β pathways, while oncogenic miRNAs drive angiogenesis and growth signals. Ultimately, five novel regulatory miRNAs including hsa-miR-618 were identified and validated (132). A study by Shefer et al. defined breast cancer exosomal proteins as “malignant functional clusters” and “endothelial overlapping clusters” through proteomics and established the priority value of blood exosomes as a source of tumor markers based on differences between clinical samples and cell line data (133). Multi-omics integration can more comprehensively reveal tumor heterogeneity and enhance the clinical relevance of classification (134, 135).

### 5.1 Computational methods for multi-omics data integration

In this context, various multi-omics integration and analytical methods have been explored. Duan et al. systematically presented MOSD, an efficient, automatically weighted, network-based multi-omics integration method, and validated its superiority in cancer subtype identification across 10 cancer types. MOSD not only enhances the clinical significance of classification (e.g., survival differences), but also deepens the understanding of tumor biological mechanisms (136). The ConcatAE and CrossAE models developed by Tong et al. integrated multi-omics data through feature concatenation and cross-modal alignment strategies, respectively. These models demonstrated superior survival prediction performance compared to single-modality approaches in TCGA breast cancer cohort, providing a paradigm for deep learning-driven novel multi-omics integration (137). He et al. systematically elucidated the pivotal role of AI-driven multi-omics integration in precision oncology, integrating multidimensional data such as genomics, imaging, and pathology. AI technologies have demonstrated significant clinical potential in early screening (e.g., CancerSEEK), precise subtyping, prognostic assessment, and immunotherapy response prediction, providing the core driving force for achieving personalized treatment (18). Choi et al. proposed the moBRCA-net deep learning framework, which utilizes self-attention modules to analyze biological associations among gene expression levels, DNA methylation, and miRNA data. Reconstructing multi-omics representations based on feature importance significantly improves the classification performance of breast cancer subtypes while maintaining model interpretability (138).

### 5.2 Biomarker discovery

Liquid biopsy, with its minimally invasive nature, overcomes the spatial and temporal limitations of tissue biopsy, demonstrating significant clinical potential for real-time tumor monitoring and early detection of drug resistance and recurrence (139). Among these, the role of miRNAs as diagnostic and prognostic markers has gradually been discovered (140). İlhan et al. found that the combined detection of serum miR-197-3p (upregulated) and miR-1236 (downregulated) demonstrated 84.2% diagnostic accuracy in early breast cancer screening, significantly outperforming traditional single biomarkers. This provides strong evidence for advancing the clinical translation of noninvasive early diagnosis of breast cancer (141). Spychalski et al. utilized EV subpopulation-specific miRNA profiles to identify a four-miRNA panel (miR-340-5p, miR-598-3p, miR-15b-5p, and miR-126-3p) across HER2+/CD24+ vesicles through LASSO screening, offering a novel liquid biopsy strategy for precise classification of BI-RADS category 4 lesions (142). Significant progress has been made in prostate cancer research. Verma et al. developed a dual-detection model based on plasma hsa-miR-133a-3p and hsa-miR-153-3p, achieving near-perfect discrimination for prostate cancer diagnosis (AUC 0.98–1.0). Its outstanding sensitivity and specificity indicate that liquid biopsy technology is promising as a potential replacement for traditional PSA testing (143). miRNAs not only have unique advantages in the diagnosis of prostate cancer, but also hold potential in cancer staging. The total circulating small RNA concentration dynamically increases during the progression of prostate cancer and its transformation into metastatic castration-resistant prostate cancer and continues to change after treatment, highlighting its clinical utility as a biomarker for dynamic disease monitoring and prognostic evaluation (144). In the field of lung cancer, the study by Wen et al. established platelet-derived hsa-miR-199b-3p as a novel non-invasive biomarker for early detection of lung adenocarcinoma. By validating hsa-let-7i-5p as a stable internal reference, it was demonstrated that platelet miRNAs may have superior clinical detection efficacy compared to exosomal miRNAs in distinguishing benign from malignant pulmonary nodules (145). The above research progress demonstrates that the miRNA analysis of liquid biopsies has evolved from single-marker detection to a new phase of multimodal, multi-source integration. It demonstrated high-precision diagnostic and dynamic monitoring capabilities in breast cancer, prostate cancer, and lung cancer, not only overcoming the limitations of traditional tissue biopsies and single serum markers but also signifying that tumor diagnosis and treatment are gradually entering a new era of precision medicine characterized by minimally invasive, real-time, and personalized approaches. With the establishment of standardized testing systems and the deep integration of multi-omics data, miRNA liquid biopsy technology is expected to become a core tool for comprehensive cancer management, spanning early screening, precise typing, and treatment efficacy monitoring throughout the entire disease course.

## 6 Treatment strategies and clinical translation prospects

miRNAs demonstrate significant potential in cancer therapy, with their molecular basis rooted in the widespread dysregulation of the miRNA expression in tumor tissues, characterized by the loss of tumor-suppressive miRNAs and the overexpression of oncogenic miRNAs, thus providing clear targets for intervention through miRNA modulation. Cancer therapeutic strategies involving miRNAs follow two core approaches: functional inhibition of oncogenic miRNAs (via antisense therapy, small-molecule inhibitors, or miRNA sponges) and functional restoration of tumor-suppressive miRNAs (through replacement therapy). Current research frontiers have shifted towards combination strategies, including the synergistic use of multiple miRNA drugs, as well as integrating miRNA therapy with traditional chemotherapy or targeted drugs. These approaches aim to enhance therapeutic efficacy and overcome drug resistance through multitarget regulation (146).

### 6.1 miRNA replacement therapy

miRNA replacement therapy is a crucial gene silencing strategy in gene therapy that regulates the expression of disease-related genes by restoring or enhancing the expression of beneficial miRNAs in the body. Although they still face challenges such as delivery and stability, they show promising potential for the treatment of various diseases (147). The research team led by Valcourt developed a “targeting-therapy integration” nano-platform by encapsulating miR-34a mimics into Notch1 antibody-functionalized nanoparticles, achieving synergistic treatment for triple-negative breast cancer: antibody-mediated targeting combined with Notch signaling inhibition, along with the tumor-suppressing function of miR-34a, collectively exerting anti-tumor effects (148). Interestingly, in lung cancer, miR-34a serves as a key tumor suppressor that regulates the TGF-β signaling pathway, acting as a central hub for maintaining cellular homeostasis, inhibiting abnormal proliferation, and preventing epithelial-mesenchymal transition (EMT). Its crucial biological functions have made it the first miRNA therapeutic agent to enter clinical trial (149, 150). Nanoparticle-based miR-143 replacement therapy demonstrates promising therapeutic effects in prostate cancer models by specifically targeting the urokinase-type plasminogen activator receptor and inhibiting its translation process, thus providing a new treatment paradigm for miRNA replacement therapy (151). Mohammadi et al. found that miR-330 is downregulated in lung cancer tissues and associated with a poor patient prognosis. Restoring its expression can achieve broad-spectrum anti-tumor effects by synergistically inducing apoptosis and inhibiting migration, highlighting its clinical potential as a candidate molecule for alternative lung cancer therapies (152).

### 6.2 Restoring tumor-suppressing miRNA

MicroRNA (miRNA) regulation has emerged as a promising field for cancer therapy. Substantial *in vivo* experimental evidence has demonstrated that restoring tumor-suppressive miRNA function exhibits significant clinical translational potential for tumor suppression, highlighting the need for the development of next-generation anticancer drugs (153). Ryu et al. developed an anti-CD47 antibody-miR-34a conjugate (aCD47-C-miR34a) targeting triple-negative breast cancer, overcoming the clinical application barriers of miR-34a through a CD47-mediated targeted delivery system. This combination therapy not only restored the tumor-suppressive function of miR-34a but also remodeled the tumor immune microenvironment by downregulating PD-L1, activating dual mechanisms of macrophage phagocytosis, and enhancing CD8+ T cell activation, achieving significant tumor suppression without systemic toxicity (154). Ahrend et al. systematically compared the effects of 2’-O-methyl and 2’-fluoro modifications on miR-1. Their research revealed that chemical modifications not only maintained their detection stability but also significantly enhanced their bioactivity in inhibiting the growth of prostate cancer cells, providing a crucial technical pathway for optimizing therapeutic miRNAs (155).

### 6.3 Inhibiting cancer-causing miRNAs

Oncogenic miRNAs play a central role in malignant processes, such as cell proliferation, metastasis, and drug resistance. Therefore, targeting and inhibiting oncogenic miRNAs to restore normal gene regulatory programs has emerged as a promising strategy for cancer treatment. The core function of oncogenic miRNAs in tumorigenesis is highlighted, with a focus on two major targeting approaches: the design of antisense oligonucleotides (e.g., AntagomiRs) to directly antagonize specific oncogenic miRNAs, and the utilization of miRNA sponge molecules to efficiently adsorb multiple miRNAs and relieve their inhibitory effects (156).

### 6.4 AntagomiRs

As an antisense oligonucleotide therapy targeting oncogenic miRNAs, the core advantage of AntagomiR lies in its ability to precisely inhibit target oncomiRs through highly specific complementary binding. Chemical modifications such as LNA significantly enhance its *in vivo* stability, affinity, and therapeutic efficacy. Several drugs have entered clinical research stages, demonstrating broad translational prospects. However, this technology also faces numerous challenges, mainly due to the low delivery efficiency *in vivo*, as naked oligonucleotides are prone to degradation and difficult for cells to effectively absorb. Additionally, they may trigger immunogenic reactions, pose potential off-target risks, and typically can only target a single miRNA, making it challenging to address the complex network of multiple miRNAs that work synergistically in cancer (157). However, these factors have limited their clinical application.

### 6.5 miRNA sponges

The core advantage of miRNA sponges is their ability to function as stably expressed competitive inhibitors, enabling long-term, highly efficient suppression of target miRNAs (even entire miRNA families sharing seed sequences or multiple distinct miRNAs). By incorporating a central bulge design, they can avoid degradation by AGO2, thereby enhancing the durability of the inhibition. However, this technology also has significant drawbacks; the plasmid construction process is highly complex and time-consuming, involving multiple steps of molecular cloning and screening. Additionally, the number of binding sites (MBS) requires fine-tuning to balance efficacy and stability, and there is a risk of potential off-target and nonspecific effects, necessitating rigorous control designs to mitigate these issues (158). Therefore, despite being a powerful research tool, it still faces delivery challenges for *in vivo* therapeutic applications.

### 6.6 Clinical translation challenges

Although miRNAs demonstrate vast potential in precision medicine, their translation from basic research to clinical applications still faces key scientific barriers such as delivery efficiency, *in vivo* stability, and targeting specificity. The research team led by Hong conducted the first human clinical trial on miRNA-targeted therapy. While they successfully demonstrated that miR-34a mimics could be delivered to tumors and achieved the intended gene regulation, providing proof of concept, clinical development was forced to terminate prematurely due to severe challenges. The core challenge lies in triggering unpredictable and fatal immune-mediated toxicities that have not yet been effectively predicted in preclinical animal models. Additionally, the therapy faces multiple dilemmas, including insufficient delivery efficiency and targeting, limited anti-tumor efficacy leading to an unfavorable risk-benefit ratio, and unclear mechanisms of action, highlighting the significant difficulty in safely translating such therapies into clinical applications (159). The anti-miR drug RG-101 targeting miR-122 demonstrated therapeutic potential in Phase I clinical trials, but its Phase II study was discontinued due to reports of unexpected severe hyperbilirubinemia cases (160). In summary, these early pioneering clinical trials successfully validated the conceptual feasibility of miRNA-targeted therapy, yet more profoundly revealed the common challenges faced in clinical translation: unpredictable immune toxicity, predictive limitations of preclinical models, and delivery systems that urgently require optimization. Future breakthroughs will depend on the development of safer delivery vectors, more precise targeting strategies, and establishment of more reliable preclinical toxicity prediction models.

### 6.6 Conclusions and outlook

In summary, multi-omics technologies have significantly advanced our understanding of the central regulatory role miRNAs play within the bone metastasis microenvironment of breast, prostate, and lung cancers. While shared microenvironmental features exist across these cancers, distinct functional and structural profiles characterize each type, influencing disease progression and therapeutic response. Multi-omics integration enables a systems-level view of miRNA-mediated regulation, highlighting their role in intercellular communication and key pathway modulation, which governs colonization, immune evasion, and bone remodeling.

Clinically, miRNAs offer promising dual utility as diagnostic biomarkers and therapeutic targets, with emerging strategies showing efficacy in preclinical models. However, challenges in delivery, stability, and model predictability remain barriers to translation. Future efforts should prioritize the development of targeted delivery systems, multi-omics-guided computational models, cross-cancer comparative studies, and clinically relevant experimental platforms.

**Figure 5:**
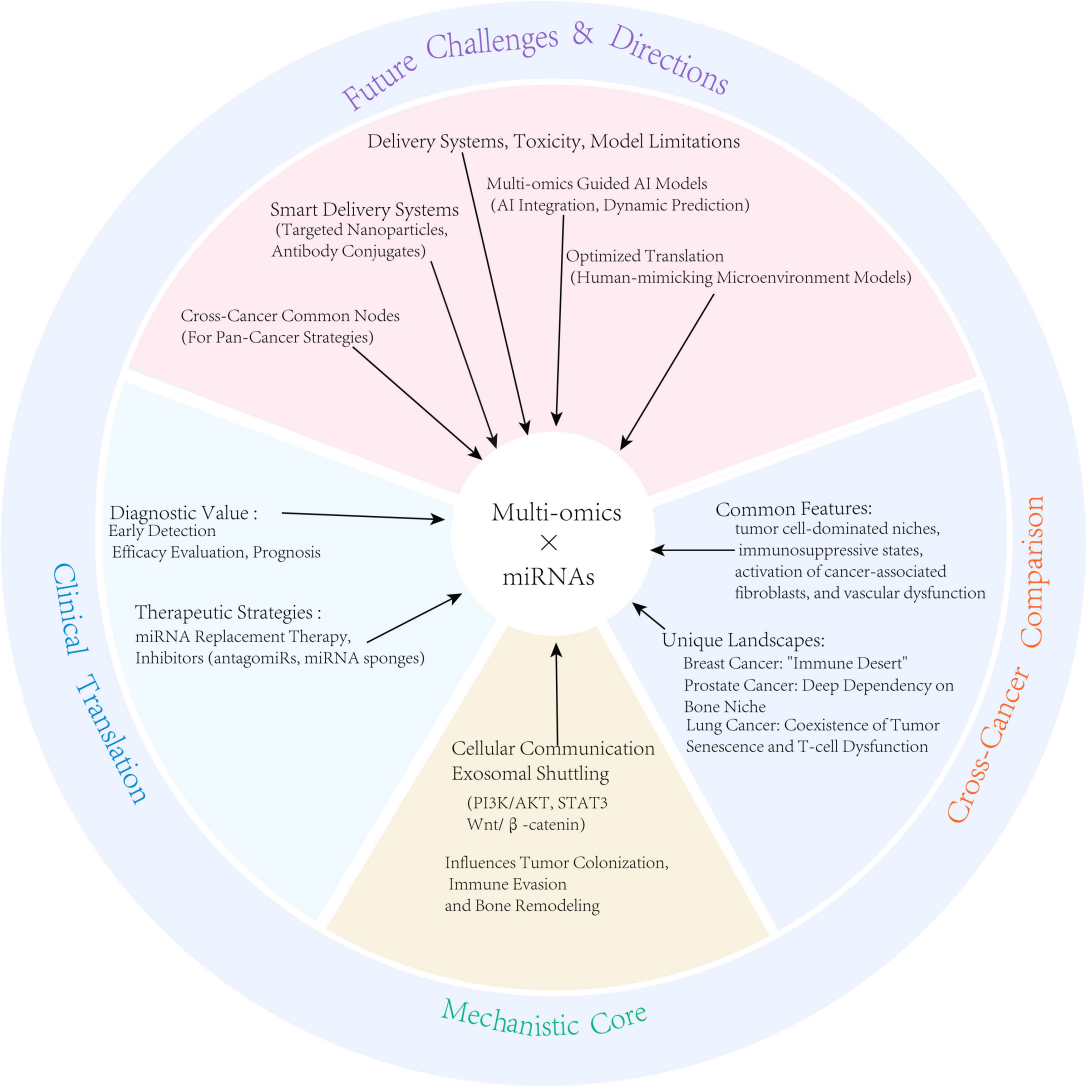
Schematic representation of the multi-omics guided miRNA regulatory network in bone metastasis.

Ultimately, the multi-omics perspective is reshaping the miRNA functional landscape in bone metastasis and paving the way for next-generation diagnostics and personalized therapies, positioning miRNAs as pivotal elements in the future of precision oncology, as shown in **Figure 5**.
